# QTLs Analysis and Validation for Fiber Quality Traits Using Maternal Backcross Population in Upland Cotton

**DOI:** 10.3389/fpls.2017.02168

**Published:** 2017-12-22

**Authors:** Lingling Ma, Yanpeng Zhao, Yumei Wang, Lianguang Shang, Jinping Hua

**Affiliations:** ^1^Laboratory of Cotton Genetics, Genomics and Breeding, College of Agronomy and Biotechnology/Beijing Key Laboratory of Crop Genetic Improvement/Key Laboratory of Crop Heterosis and Utilization of Ministry of Education, China Agricultural University, Beijing, China; ^2^Department of Cotton Breeding, Institute of Cash Crops, Hubei Academy of Agricultural Sciences, Wuhan, China

**Keywords:** fiber quality, stable QTL, backcross population, Upland cotton, epistasis detection

## Abstract

Cotton fiber is renewable natural fiber source for textile. Improving fiber quality is an essential goal for cotton breeding project. In present study, F_14_ recombinant inbred line (RIL) population was backcrossed by the maternal parent to obtain a backcross (BC) population, derived from one Upland cotton hybrid. Three repetitive field trials were performed by randomized complete block design with two replicates in three locations in 2015, together with the BC population, common male parent and the RIL population. Totally, 26 QTLs in BC population explained 5.00–14.17% of phenotype variation (PV) and 37 quantitative trait loci (QTL) were detected in RIL population explaining 5.13–34.00% of PV. Seven common QTLs detected simultaneously in two populations explained PV from 7.69 to 23.05%. A total of 20 QTLs in present study verified the previous results across three environments in 2012. Particularly, *qFL-Chr5-2* controlling fiber length on chromosome 5 explained 34.00% of PV, while *qFL-Chr5-3* only within a 0.8 cM interval explained 13.93% of PV on average in multiple environments. These stable QTLs explaining great variation offered essential information for marker-assisted selection (MAS) to improve fiber quality traits. Lots of epistasis being detected in both populations acted as one of important genetic compositions of fiber quality traits.

## Introduction

Upland cotton (*Gossypium hirsutum* L.) is one of the most important fiber crops, which accounts for more than 92% of cultivated cotton worldwide because of its higher yield potential, and adaptation to diverse environments (Zhang T. et al., 2015). But fiber quality of Upland cotton is not as good as that of Sea Island cotton (*G. barbadense* L.). Because of the fiber quality traits are quantitative traits and highly affect by genetic background and growing environment. Therefore, it is an important target to improve fiber quality in cotton breeding programs.

Recombinant inbred line (RIL) population has been considered as a permanent mapping population, in which the number of homozygous alleles could be used to dissect the additive effect of allele(s). The backcross (BC) population deriving from RIL population by test cross or back cross, is suitable to be used to identify QTLs, to detect the dominant effect, and to carry out multiple trials across multi-environments and repeat observations, similarly to some extent to “immortalized” F_2_ population (Hua et al., 2002, 2003; Mei et al., 2005; Shang et al., 2016a). Several studies had been performed in other crops in yield and yield-component traits using BCF_1_ segregation populations based on RIL populations (Xiao et al., 1995; Mei et al., 2005; Larièpe et al., 2012).

Some interspecific BC_1_F_1_ populations were developed from *G. hirsutum* × *G. barbadense* for fiber quality traits QTL mapping in cotton (Said et al., 2015). For example, seven QTLs related to fiber length and fiber strength were detected using BCF_1_ segregating population deriving from Guazuncho 2 × VH8-4602 (Lacape et al., 2005), 44 QTLs for fiber quality traits were detected using (CCRI-8 × Pima 90-53) × CCRI-8 BCF_1_ interspecific population, especially including four QTLs locating on chromosome (Chr) 5 (Yang et al., 2015). A meta-analysis including 728 QTLs for fiber quality traits showed that QTL-rich regions on Chr 5, Chr 7, Chr 14, Chr 19, Chr 21, and Chr 26 were related to fiber length, fiber strength and Micronaire, and 11 QTLs for fiber length and fiber strength were distributed on Chr 21, respectively (Said et al., 2013). The majority of QTLs have also been mapped using intra-species RIL populations of Upland cotton (Wang et al., 2006; Shen et al., 2007; Wan et al., 2007; Wu et al., 2009; Sun et al., 2012; Ning et al., 2014; Tan et al., 2014; Shang et al., 2015, 2016a,b,c; Tang et al., 2015; Zhang J. et al., 2015; Zhang Z. et al., 2015; Jamshed et al., 2016; Li et al., 2016). Recently, intraspecific BCF_1_ populations in Upland cotton were constructed to map QTLs based on backcross of RIL population to the parental lines, respectively (Shang et al., 2016a; Wang et al., 2016). Shang et al. (2016d) detected 62 stable QTLs related to fiber quality traits in F_9_ RIL populations and their maternal BCF_1_ progenies. Wang et al. (2016) used two parental BCF_1_ populations and mapped four, one and three stable QTLs in more than one environment(s) or population(s) for fiber length, fiber strength, and Micronaire, respectively.

The genome (2.5 Gb) of allotetraploid Upland cotton harbors 26 chromosomes and displays asymmetric evolution between the A subgenome (A01–A13) and the D subgenome (D01–D13) (Zhang T. et al., 2015). The rapid development of genome sequencing technology facilitated the availability of cotton genomic data of diploid species A_2_ and D_5_ (Paterson et al., 2012; Wang K. et al., 2012; Li et al., 2014), and tetraploid genomes (AD)_1_ and (AD)_2_ (Li et al., 2015; Liu et al., 2015; Yuan et al., 2015; Zhang T. et al., 2015), which provided new insights to the development of molecular markers; from microsatellite (SSR) markers to single nucleotide polymorphism (SNP) markers and other valuable resources in breeding projects. Recently, a genetic map with 2618 SNP loci was constructed by genotyping with cotton 63K SNP array (Hulse-kemp et al., 2015). Nine stable QTLs related to fiber quality traits across two environments were detected, and one fiber length hotspot on Chr 5 carrying three QTLs was observed (Li et al., 2016). Four potential candidate genes for fiber length on Chr Dt7 were found using a total of 81,675 SNP markers genotyping by sequencing through genome-wide association studies (GWAS) (Su et al., 2016). The development of genome re-sequencing researches offered new insights to cotton genome (Fang et al., 2017; Wang M. et al., 2017). It is necessary to verify QTLs accurately under maximum environments and more segregation populations, especially stable QTLs across multiple environments. Because of the importance of cotton fiber quality, it is essential to dissect the genetic mechanism of fiber quality on Upland cotton using diverse design populations, including intraspecific BCF_1_ segregation population.

In our previous study, segregating populations including F_2:3_, F_2:4_, RIL, and BCF_1_ population derived from the same hybrid “Xinza 1” had been developed and used to dissect the genetic effects for fiber quality traits (Liang et al., 2013; Shang et al., 2015, 2016d) and yield-related traits (Liang et al., 2015; Shang et al., 2016a,b,c). Markers of MUSS193-Gh388 flanking the common *qFL-Chr5-2* could be further validated its role in markers assisted selection (MAS), which controlled fiber length on Chr 5 and explained 20.12 and 14.77% of PV across two locations (Liang et al., 2013). In addition, the region of SWU15194-HAU190 on Chr 9 was detected for Micronaire by two times and explained highly to 25.93% of PV; another region of PGML1917-SWU17715 on Chr 5 increased 0.29 mm fiber length with 21.09% of phenotypic variance explained (PVE); Region of NAU4034-SWU17713 on Chr 5 controlled fiber length, fiber strength, and fiber elongation at the same time, in which, a stable QTL namely *qFE-Chr5-1* explained 20.41% of PV (Shang et al., 2016d). In order to resolve genetic components related fiber quality traits and reveal the genetic mechanism more details, we further developed BCF_1_ progenies population based on RIL population by testcrossing with the maternal parent and attempted to investigate QTLs in fiber quality traits in both RIL and BCF_1_ populations across three environments in present study.

## Materials and methods

### Plant materials and population development

The F_14_ RIL population derived from the Upland cotton hybrid “Xinza 1” (GX 1135 × GX 100-2) was employed, which was developed by single seed descent method (Shang et al., 2016a). Two experimental populations were deduced based on the design: (1) backcross population, comprising 177 BCF_1_ progenies, was developed by testcrossing 177 F_14_ RILs with maternal parent GX1135, respectively. (2) RIL population, including 177 lines of F_15_ generations.

The control set (GX1135, “Xinza 1” F_1_, GX100-2, and a variety used as competition control) was performed in three field trials. The competition control in Yellow River Region (2015E1 and 2015E2, see details below) was a commercial hybrid “Ruiza 816” F_1_ by cross of 087 × 884, another in Yangtze River Region (2015E3, see detail below) was a commercial hybrid “Ezamian 10” F_1_ by cross of Tai 96167 × (GK19 chosen by Tai D-3).

### Field experiment and trait evaluation

Three field trials based on RIL population and maternal BC population were conducted in 2015E1, 2015E2, and 2015E3, E1: Quzhou Experimental Station of China Agricultural University, Handan City, Hebei Province (36°78′N, 114°92′E) in Yellow River valley; E2: Guoxin Seed Company Ltd, Cangzhou City, Hebei Province (38°43′N, 116°09′E) in Yellow River valley; E3: Hubei Academy of Agricultural Sciences, Wuhan City, Hubei Province (30°29′N, 114°19′E) in Yangtze valley. In addition, previous study in 2012 performed three same trials in E1 (2012E1) in Handan City, E2 (2012E2) in Cangzhou City, and E4 (2012E4) in Xiangyang City, Hubei Province (30°18′N, 112°15′E) (Shang et al., 2016a,d).

Seedlings were cultured on May 14th and May 15th (E1) and on April 19th, 2015 (E3). The transplanting was on June 12th (20–37°C) and June 13th (20–34°C), 2015 (E1), and on May 9th (17–28°C) and May 10th (15–29°C), 2015 (E3), respectively. Seeds were directly sowed on April 25th and 26th (17–31°C), 2015 (E2). The climate and weather in 2015 (E1, E2, and E3) was regular during cotton growth season, excepting that one trial encountered hailstone one June 11th, 2015 (E2). Since all the experiment materials were under the identical environment so we strengthened field managements in time to recover the seedling development after hailstone disaster (2015E2) so as to obtain the data sets. The flowering time had been postponed 6 weeks in that environment comparing with normal.

The field trials were planted following a randomized complete block design with two replications each trial, which included 904 plots with two rows per plot (22 individuals each). For one replication, 177 BCF_1_ progenies were inter-planted in the middle of 177 RI Lines as their maternal parents and the common testing-male parent GX1135 (original maternal parent), together with two control sets (GX1135, “Xinza 1” F_1_, GX100-2, control: “Ruiza816” F_1_ at E1 and E2, or “Ezamian 10” F_1_ at E3 and E4). Two-row plots were 90 and 50 cm row spacing alternately for the experiment in E1 and E2, and two-row plots in E3 were 100 and 50 cm row spacing alternately. One plot was on the length of 3.0, 3.0, and 2.4 m respectively at 2015E1, 2015E2, and 2015E3, following 0.7 m pavement apart. Field management followed the local conventional standard field practices.

Twenty-five naturally opening bolls in the middle of plants were hand-harvested for each plot at mature stage in three environments, respectively. Fiber samples were ginned and sampled for measurements of fiber quality traits with HVI 900 instrument (USTER_ HVISPECTRUM, SPINLAB, USA) at Cotton Fiber Quality Inspection and Test Center of Ministry of Agriculture (Anyang, China). The fiber quality traits included fiber length (mm), fiber uniformity (%), fiber strength (cN/tex), fiber elongation, and Micronaire. The total sample numbers were 892, 904, and 884 at 2015E1, 2015E2, and 2015E3, respectively.

### Genetic map and data analysis

The genetic map based on RIL population published before by screening polymorphic loci between two parents using 48,836 pairs of SSR primer (Shang et al., 2016d). The source of the SSR markers contained types of BNL, NAU, TM, JESPER, CIR, HAU, CM, MUSS, MUSB, MUCS, SWU, PGML, and CAU from *Gossypium*, which can be obtained from Cotton Microsatellite Database (http://www.cottonmarker.org) and CottonGen Database (https://www.cottongen.org) (Liang et al., 2013; Shang et al., 2016d). A total of 623 loci distributed on 31 linkage groups and anchored on 26 chromosomes, covering 3889.9 cM (88.20%) of cotton genome with average interval of 6.2 cM. The genotype for each maternal BCF_1_ was deduced on the basis of the RIL genotype used as the parent for testcross (Shang et al., 2016a,b,c).

Basic statistical analysis was implemented by the software SPSS (Version 19.0, SPSS, Chicago). The software QTL Cartographer (Version 2.5) was used to map single-locus QTL and to estimate the genetic effect (Zeng, 1994; Wang S. et al., 2012). The composite interval mapping (CIM) method was chosen for QTL mapping in the confidence interval of 95%. The precision selected 1.0 cM walk speed. The threshold of LOD 3.0 was used to declare a suggestive QTL after performing a 1,000 permutation test, whereas QTLs in another environment or population with at least LOD 2.5 were considered as common QTL (Liang et al., 2013; Wójcik-Jagła et al., 2013; Shang et al., 2015, 2016d). According to the position linked and shared common markers, QTLs detected in different populations were regarded as common QTL (Shao et al., 2014). The QTL IciMapping 4.1 (www.isbreeding.net) was conducted by the two-locus analysis using inclusive composite interval mapping (ICIM) method. A threshold LOD 2.5 and LOD 5 scores were used to declare significant M-QTL and E-QTLs, respectively. Mapping by IciMapping 4.1, the main-effect QTL (M-QTL) and its environmental interaction (QTL × environment, QE), epistatic QTLs (E-QTLs), and its environmental interactions (QTLs × environment, QQE) were conducted using RIL and BC datasets under three environments.

## Results

### Trait performance of parents and two populations

The performances of “original” maternal parent “GX1135” were superior to those of “original” male parent “GX100-2” for fiber length (FL) in three locations, as well as for fiber uniformity (FU), fiber strength (FS), and fiber elongation (FE) in 2015E2 and 2015E3 (Table 1). On the contrary, performance of GX100-2 was superior to performance of GX1135 for Micronaire (FM) in three environments. Phenotype performance of five fiber quality traits varied in three environments such as that fiber quality in 2015E1 was always inferior to fiber quality in 2015E2 and 2015E3 (Table 1). It attributed to the diverse photo-thermal conditions, fertilizer application and growth regulators over the development stages in diverse environments. “Xinza 1” displayed −0.63% mid-parent heterosis (MPH, %) on average for five fiber quality traits (Table 1) and MPH (%) in BC population ranged from 0.08 to 2.91% among 177 BCF_1S_, in accordance with the previous study (Tables S1, S7) (Shang et al., 2016d). The result demonstrated that there was no significant hybrid vigor for fiber quality traits.

**Table 1:** Summary statistics on fiber quality trait data of RIL and BC populations in three environments.

**Trait**	**Env.^a^**	**BC**^**b**^			**RIL**			**Parents**				
		**Mean**	**Range**	**CV%^c^**	**Mean**	**Range**	**CV%**	**♀**	**♂**	**Xinza 1**	**MPH%^d^**	**CK^e^**
Fiber length (mm)	2015E1	29.51	27.20–31.80	2.88	29.42	27.10–32.70	3.52	28.38	28.27	27.93	−1.39	29.40
	2015E2	31.01	28.85–33.20	2.37	30.85	28.05–33.45	3.53	30.63	30.00	30.88	1.86	31.18
	2015E3	30.91	29.10–33.20	2.51	30.77	27.85–34.05	3.53	30.30	29.85	29.73	−1.15	30.53
Fiber uniformity	2015E1	84.19	81.45–86.35	1.19	84.02	80.60–86.55	1.19	83.58	85.30	84.43	−0.01	84.93
	2015E2	85.99	83.45–87.85	0.78	85.81	83.55–87.40	0.89	86.30	85.73	86.25	0.27	86.08
	2015E3	85.09	82.30–87.05	0.94	84.95	82.15–87.00	1.08	86.50	85.48	86.00	0.01	85.70
Fiber strength (cN/tex)	2015E1	30.04	26.80–34.30	4.28	30.12	26.15–33.85	5.60	29.68	30.47	28.25	−6.07	30.67
	2015E2	30.43	27.80–34.20	3.32	30.49	27.05–36.20	4.74	30.45	29.57	30.05	0.13	31.93
	2015E3	31.23	28.15–33.80	3.84	31.18	27.10–35.60	5.24	30.20	30.18	29.45	−2.45	29.70
Fiber elongation	2015E1	6.96	6.70–7.20	1.18	6.94	6.65–7.25	1.63	6.80	6.93	6.88	0.22	6.87
	2015E2	6.98	6.80–7.20	0.94	6.96	6.70–7.15	1.21	6.98	6.93	7.00	0.65	6.98
	2015E3	7.02	6.75–7.20	1.21	7.00	6.70–7.30	1.56	6.98	6.95	6.88	−1.22	6.90
Micronaire	2015E1	4.79	3.80–5.50	6.29	4.62	3.40–5.40	8.35	4.90	4.53	4.98	5.62	5.07
	2015E2	4.91	4.10–5.70	5.69	4.85	3.95–5.80	7.31	5.15	4.87	4.78	−4.59	5.28
	2015E3	5.47	4.90–5.90	3.23	5.39	4.50–5.95	5.33	5.58	5.53	5.48	−1.35	5.40

a *Environment in 2015, E1, Handan, E2, Cangzhou, E3, Wuhan*.

b *RIL, the recombinant inbred line population; BC, the maternal backcross population*.

c *Coefficient of variation*.

d *Mid-parent heterosis (%)*.

e *Competition control of “Ruiza 816” in 2015E1 and 2015E2 and “Ezamian 10” in 2015E3. Hereinafter same*.

In RIL and BC populations, genotype variance and environment variance for five traits showed significant at level of 0.01 except FU in BC population (Table 2). Then, coefficient of variation (CV) ranged from 2.37 to 8.35% for FL, FS and FM in both populations. And, lighter phenotypic variation presented with 0.78–1.63% of CV for FU and FE in both populations. For five fiber quality traits, the majority of mean values in BC population were bigger than mean values in RIL population (Table 2). It indicated that phenotype performance decided by the performance of both parents. Heritability decreased from 0.93 in RIL population to 0.81 in BC population for fiber length and similar tendency between both populations was observed for other four traits. The results indicated that environment affected stronger for phenotype in BC population than that in RIL population. Taken together, wider ranges, bigger CV, and higher heritability for five fiber quality traits were observed in RIL population than that in BC population (Tables 1, 2).

**Table 2:** ANOVA analysis and heritability for fiber quality traits in BC and RIL populations.

		**BC**		**RIL**	
**Trait**	**Source of variation^a^**	**MS^†^**	***H*^2^^§^**	**MS**	***H*^2^**
Fiber length	G	1.97^**^	0.81	5.16^**^	0.93
	E	239.50^**^		210.47^**^	
	G × E	0.84		0.78	
	Error	1.04		0.89	
Fiber uniformity	G	1.51	0.70	2.26^**^	0.78
	E	273.51^**^		261.09^**^	
	G × E	1.27		1.20	
	Error	1.31		1.41	
Fiber strength	G	4.66^**^	0.84	10.73^**^	0.92
	E	125.46^**^		95.49^**^	
	G × E	1.67		1.94	
	Error	2.05		2.10	
Fiber elongation	G	0.87^**^	0.78	0.87^**^	0.90
	E	49.15^**^		48.80^**^	
	G × E	0.67		0.20	
	Error	0.63		0.17	
Micronaire	G	11.63^**^	0.80	30.95^**^	0.84
	E	202.39^**^		245.78^**^	
	G × E	3.73		4.84	
	Error	3.41		4.74	

a *G, Genotype; E, environment; G × E, genotype × environment*.

† *Mean square*.

§ *Heritability*.

** *indicated the significance at 0.01 probability level, respectively*.

### Correlation analysis

Correlation analysis on five fiber quality traits were performed using the mean phenotypic values both in RIL and BC populations in 2015E1, 2015E2, and 2015E3, respectively (Table 3). For fiber length, the significant and positive correlation existed with fiber strength and fiber elongation, but negatively with Micronaire in three locations in RIL and BC populations. Fiber strength displayed positive correlation with fiber elongation but negative correlation with Micronaire. The correlation showed significant and positive between fiber uniformity and fiber strength in RIL population, as well as between fiber uniformity and fiber elongation. The results showed similar tendency among five fiber quality trait in comparison with previous results (Liang et al., 2013; Shang et al., 2015, 2016d). In BC population, fiber uniformity positively correlated with fiber elongation.

**Table 3:** Correlation analysis between fiber quality traits in RIL population and its BC population.

**Trait**	**Env**.	**Fiber length**		**Fiber uniformity**		**Fiber strength**		**Fiber elongation**	
		**RIL**	**BC**	**RIL**	**BC**	**RIL**	**BC**	**RIL**	**BC**
Fiber uniformity	2015E1	0.405^**^	0.394^**^						
	2015E2	0.201^**^	0.222^**^						
	2015E3	0.236^**^	0.105						
Fiber strength	2015E1	0.652^**^	0.622^**^	0.367^**^	0.252^**^				
	2015E2	0.715^**^	0.699^**^	0.162^*^	0.075				
	2015E3	0.754^**^	0.651^**^	0.287^**^	0.146				
Fiber elongation	2015E1	0.605^**^	0.578^**^	0.361^**^	0.258^**^	0.624^**^	0.617^**^		
	2015E2	0.615^**^	0.476^**^	0.343^**^	0.163^*^	0.517^**^	0.533^**^		
	2015E3	0.782^**^	0.813^**^	0.323^**^	0.152^*^	0.728^**^	0.736^**^		
Micronaire	2015E1	−0.211^**^	−0.403^**^	0.085	−0.022	−0.216^**^	−0.372^**^	0.071	−0.109
	2015E2	−0.378^**^	−0.334^**^	0.151^*^	0.049	−0.492^**^	−0.390^**^	−0.164^*^	0.047
	2015E3	−0.535^**^	−0.357^**^	0.010	−0.022	−0.421^**^	−0.276^**^	−0.235^**^	−0.206^**^

*, ** *indicated the significance at 0.05 and 0.01 probability levels, respectively*.

### Single locus QTL analysis

**Figure 1:**
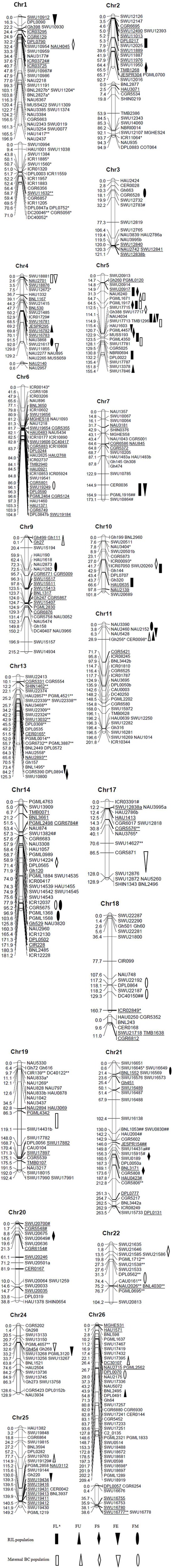
Locations of QTLs for fiber quality traits identified in RIL and BC populations. ^*^ and ^**^ (^#^ and ^*##*^), marker showed, respectively, segregation distortion significant at *P* = 0.05 and 0.01 levels; markers with ^*^ and ^**^ skewed toward the GX1135 alleles, and markers with ^#^ and ^*##*^ skewed toward the GX100-2 alleles. ^a^FL, fiber length; FU, fiber uniformity; FS, fiber strength; FE, fiber elongation; FM, Micronaire.

In three field trials, a total of 55 QTLs were detected in two corresponding populations at 2015E1, 2015E2, and 2015E3, with 5.00–34.00% of phenotypic variance explained (PVE) (Figure 1, Table 4). In our previous study, 71 QTLs were detected in the same maternal BC and same RIL population for fiber quality traits in 2012E1, 2012E2, and 2012E4 (Shang et al., 2016d). Here, 20 QTLs appearing both in 2015 in this study and in 2012 in previous study were showed in Table S2. These QTLs detected repeatedly in both years, were anchored on 21 chromosomes, respectively.

**Table 4:** QTLs detected by single-locus analysis in RIL and BC populations.

**Traits**	**QTL**	**Env.^a^**	**Flanking markers**		**BC**			**RIL**		
					**LOD**	**Effect value^b^**	**Var(%)^c^**	**LOD**	**Effect value**	**Var(%)**
Fiber length	*qFL-Chr2-1*	2015E3	DPL0217	SWU12025				3.00	0.26	5.65
	*qFL-Chr4-1*	2015E3	SWU18881	NAU2701	2.87	0.20	6.09			
	***qFL-Chr5-1**^†^*	2015E2	SWU20917	NAU6240				2.72	0.30	7.47
		2015E2	NAU6240	PGML1671	2.83	0.24	10.35			
		2015E3	NAU6240	PGML1671				4.63	0.35	10.11
	***qFL-Chr5-2***	2015E1	PGML1917	SWU17715				6.83	0.61	34.00
		2015E2	PGML1917	SWU17715	4.78	0.28	14.07			
	***qFL-Chr5-3***	2015E1	Gh388	SWU17713				10.22	0.47	19.63
		2015E2	Gh388	SWU17713				5.39	0.36	10.65
		2015E2	SWU17713	HAU1603	5.62	0.25	11.52			
	***qFL-Chr5-4***	2015E2	PGML4350	SWU17781	3.89	0.22	8.99			
		2015E3	MUSS193	PGML4350				4.44	0.32	8.55
	*qFL-Chr5-5*	2015E3	NBRI0694	DPL0022				3.66	0.29	7.13
	*qFL-Chr7-1*	2015E3	CER0036	PGML1916				3.17	−0.33	8.98
	*qFL-Chr10-1*	2015E1	SWU20260	Gh144				3.15	−0.25	5.61
	*qFL-Chr10-2*	2015E1	Gh320	HAU0635				3.60	−0.33	10.29
	*qFL-Chr19-1*	2015E2	PGML4342	SWU14431b	2.76	0.25	11.16			
Fiber uniformity	*qFU-Chr4-1*	2015E3	JESPR295	SWU16782				3.10	−0.24	6.58
	*qFU-Chr5-1*	2015E3	NAU6240	PGML1671	2.74	0.26	9.90			
	*qFU-Chr5-2*	2015E1	Gh388	SWU17713				7.23	0.39	14.53
	*qFU-Chr5-3*	2015E1	SWU13378	SWU17846				3.96	−0.29	7.62
	*qFU-Chr9-1*	2015E2	Gh111	Gh27	2.54	0.16	5.15			
	***qFU-Chr11-1***	2015E1	NAU5428	Gh256	2.62	−0.25	5.97			
		2015E3	NAU5428	Gh256				2.74	−0.29	9.51
	*qFU-Chr25-1*	2015E3	SWU19763	SWU19129	2.62	−0.19	5.63			
	*qFU-Chr26-1*	2015E2	SWU17395	DC30107	2.76	0.16	5.64			
	*qFU-Chr26-2*	2015E2	BNL2495	DPL0491				2.52	0.20	5.57
Fiber strength	*qFS-Chr1-1*	2015E2	DPL0790	SWU10954	2.52	−0.23	5.00			
	*qFS-Chr2-1*	2015E2	SWU11950	TMB1268				3.85	0.40	7.41
	*qFS-Chr5-1*	2015E2	SWU20917	NAU6240				3.19	0.43	8.85
	***qFS-Chr5-2***	2015E1	PGML1917	SWU17715				4.79	0.92	29.65
		2015E2	SWU17715	Gh388	2.85	0.26	6.00			
		2015E2	Gh388	SWU17713				2.72	0.33	5.13
	*qFS-Chr5-3*	2015E1	PGML4457	MUSS193				3.37	0.46	7.16
	*qFS-Chr10-1*	2015E2	ICR00093	ICR07050	2.74	−0.25	5.72			
	*qFS-Chr13-1*	2015E1	DPL0894	SWU10800				2.80	0.40	5.60
	*qFS-Chr14-1*	2015E1	SWU14224	DPL0565	2.74	−0.32	5.99			
	*qFS-Chr20-1*	2015E3	SWU20675	SWU20649				3.14	−0.45	7.14
	*qFS-Chr21-1*	2015E2	SWU15915	SWU0189				3.28	−0.37	6.35
	*qFS-Chr21-2*	2015E3	BNL3171	CGR5808				4.44	−0.51	9.65
	*qFS-Chr22-1*	2015E2	SWU21646	SWU21585	3.17	0.26	6.29			
Fiber elongation	*qFE-Chr1-1*	2015E2	SWU10912	DPL0090				4.13	0.03	12.51
	*qFE-Chr4-1*	2015E3	SWU18881	NAU2701	2.57	0.02	5.68			
	*qFE-Chr4-2*	2015E1	SWU21617	SWU11855	3.21	−0.03	12.41			
	*qFE-Chr5-1*	2015E1	Gh388	SWU17713				7.17	0.04	14.23
	***qFE-Chr5-2***	2015E1	PGML4457	MUSS193				5.37	0.04	11.43
		2015E3	HAU1603	PGML4457				3.72	0.03	8.77
	*qFE-Chr7-1*	2015E3	CER0036	PGML1916				2.72	−0.03	8.23
	*qFE-Chr11-1*	2015E2	NAU2152	NAU5428				3.66	−0.02	7.19
	*qFE-Chr13-1*	2015E1	BNL1495	CGR5390				2.63	0.03	5.98
	*qFE-Chr17-1*	2015E2	CGR5871	SWU12876	4.10	−0.02	10.40			
	***qFE-Chr24-1***	2015E2	PGML4657	Gh454	2.58	−0.02	6.17			
		2015E2	Gh454	SWU13268				3.17	−0.02	6.03
	*qFE-Chr25-1*	2015E2	Gh220	SWU19434				2.94	0.02	6.10
Micronaire	*qFM-Chr3-1*	2015E1	Gh663	CGR6528				2.74	−0.10	5.92
	*qFM-Chr4-1*	2015E2	NAU3868	SWU21617				2.76	0.09	5.67
	*qFM-Chr6-1*	2015E1	CGR5801	SWU19249	4.17	−0.09	9.05			
	*qFM-Chr9-1*	2015E3	NAU2873	NAU1282				2.77	0.07	5.70
	*qFM-Chr11-1*	2015E2	NAU3390	NAU2460				3.02	0.09	6.15
	***qFM-Chr14-1***	2015E1	ICR12037	CGR5675	3.59	0.11	11.81			
		2015E3	ICR12037	CGR5675				2.57	0.09	9.25
	***qFM-Chr14-2***	2015E2	PGML1368	PGML1568				3.14	0.09	6.67
		2015E3	PGML1568	Gh529				3.20	0.09	10.38
	*qFM-Chr18-1*	2015E3	SWU22192	DPL0864	2.73	0.04	5.69			
	*qFM-Chr18-2*	2015E3	SWU22187	DC40150	3.23	0.05	8.96			
	*qFM-Chr21-1*	2015E1	SWU16649	BNL1552				2.68	0.12	8.72
	*qFM-Chr22-1*	2015E3	DPL0562	CAU0161	2.99	0.05	6.32			
	*qFM-Chr26-1*	2015E3	DPL0070	NAU2175	2.64	−0.04	5.94			

a *Environment, 2015E1: Handan, 2015E2: Cangzhou, 2015E3: Wuhan*.

b *The genetic value of a detected QTL, which is the additive effect estimated from the RILs mean values, the additive and dominance effects from female BC mean values*.

c *Phenotypic variation explained*.

† *Bold figures indicated the stable QTL detected in more than one environment or population simultaneously*.

For fiber length, six and 9 QTLs were identified in the BC and the RIL populations, respectively (Figure 1, Table 4). Of these, six QTLs showed positive additive effects originated from GX1135 alleles and three QTLs increased fiber length through offering alleles by GX100-2 in RIL population. Five QTLs (45.45%) were distributed on Chr 5. Four stable QTLs, *qFL-Chr5-1, qFL-Chr5-2, qFL-Chr5-3*, and *qFL-Chr5-4*, were simultaneously identified in the RIL and BC populations across multiple environments. The *qFL-Chr5-2* explained 14.07–34.00% of phenotypic variance (PV, hereinafter same). Nine (82.82%) QTLs, located on Chr 2, Chr 5, Chr 9, and Chr 11, were verified the previous results in 2012 (Table S2). The *qFL-Chr5-3* explained highly to 19.63% of PV under multiple environments in both populations, within a 0.8 cM interval flanked by SSR markers Gh388 and SWU17713. In recent study, one fine mapping QTL named *qFL-Chr1* was most likely located in a 0.9 cM interval flanked by the SSR markers MUSS422 and CIR018 (Xu et al., 2017). For five stable QTLs detected in both populations in present study, the values of genetic effects reduced in the BC population, while homozygotes became heterozygotes in each BCF_1_ following backcrossing to GX1135. Ignoring the effect by environment, additive effects of homozygotes contributed more than dominant effects of heterozygotes for fiber length, such as that *qFL-Chr5-2* increased 0.61 mm for fiber length in 2015E1 in RIL population whereas it was 0.28 mm in 2015E2 and 0.29 mm in 2012E1 in BC population (Table S2).

Nine QTLs for fiber uniformity were identified. Of those, three QTLs (*qFU-Chr5-1, qFU-Chr5-2, qFU-Chr5-3*) were anchored on Chr 5, explained 9.90, 14.53, and 7.62% of PV, respectively. One stable QTL (*qFU-Chr11-1*) was detected at 2015E1 in maternal BC population and at 2015E3 in RIL population.

For fiber strength, a total of 12 QTLs were detected (Table 4). Four QTLs were detected at least in two environments and in 2 years (Table S2). One stable QTL (*qFS-Chr5-2*) was observed in two environments and both populations, explaining 5.13, 6.00, and 29.65% of PV, respectively. On Chr 5, the flanking markers of *qFS-Chr5-1* and *qFS-Chr5-2* were same to that of *qFL-Chr5-1* and *qFL-Chr5-3*, respectively, suggesting the pleiotropic effects on improving fiber quality in Upland cotton. One QTL near the two region increased 0.33 mm fiber length and 0.49 cN/tex fiber strength on average. The genetic effects of the QTLs from RI lines were stronger than that from the corresponding BCF_1_s, whatever QTLs were detected on Chr 5 or on Chr 21. It indicated that additive QTLs played more important role in improving fiber length and fiber strength.

Eight and four QTLs for fiber elongation were identified in the RIL and BC populations, respectively. Two QTLs (*qFE-Chr5-1, qFE-Chr5-2*) were confirmed by previous result in 2012 under multiple environments and populations. Five QTLs explained PV over 10% involving in *qFE-Chr1-1, qFE-Chr4-2, qFE-Chr5-1, qFE-Chr5-2*, and *qFE-Chr17-1*. The phenotypic variation explaining by *qFE-Chr5-1* reached to 14.23% and *qFE-Chr4-2* explained 12.41% of PV (Table 4). We also observed a pleiotropic region (NAU4034-SWU17713) on Chr 5. Two stable QTLs (*qFE-Chr5-2, qFE-Chr24-1*) from RIL population were verified in BC population.

A total of 12 QTLs underlying Micronaire were located on 9 chromosomes (Table 4), in which five QTLs were detected repeatedly in 2012 (Table S2), and seven QTLs distributed on At-subgenome and five on Dt-subgenome, respectively. Two stable QTLs (*qFM-Chr14-1* and *qFM-Chr14-2*) were detected across two environments, explained 11.81 and 10.38% of PV on average, respectively. The *qFM-Chr9-1* flanking with SWU15194-HAU190 was validated a common QTL explaining highly to 25.93% in 2012E4 in the previous result (Table S2).

On summary, 37 QTLs existed in RIL population with on average 9.43% of PVE; and 26 QTLs in BC population with average 7.92% of PVE (Figure 1, Table 4). The number of QTLs in the RIL population was always more than that in the BC population, which showed similar tendency with the previous conclusion in 2012 (Shang et al., 2016d). In present study, a total of 10 stable QTLs were detected across two populations or in at least two environments. Of these, eight QTLs were detected in both populations: *qFL-Chr5-1, qFL-Chr5-2, qFL-Chr5-3, qFL-Chr5-4, qFU-Chr11-1, qFS-Chr5-2, qFE-Chr24-1*, and *qFM-Chr14-1*. A co-location between Gh388 (SWU17717, NAU4034) and TMB1296 (SWU17713) contained four QTLs (*qFL-Chr5-3, qFS-Chr5-2, qFE-Chr5-1, qFU-Chr5-2*) controlling fiber length, fiber strength, fiber elongation, and fiber uniformity together. The pleiotropic region was consistent with the region of *qFL-Chr5-4, qFS-Chr5-2, qFE-Chr5-1* in previous study (Shang et al., 2016d).

### QTLs by environment interactions and digenic interactions

Totally, 102 M-QTLs and environmental interactions (QTL × environment, QE) were identified (Table 5, Tables S3–S6). Of these, 62 and 38 M-QTLs and QEs existed in the RIL population and its BC population, explained 0.13–5.95 and 0.12–7.61% of PV (Tables S3, S5), respectively. Environmental effect prevailed in both populations, but it affected M-QTLs in the BC population in a greater extent than that in RIL population. Then, 97 and 67 E-QTLs and their environmental interactions (digenic interactions × environment, QQE) were respectively identified in RIL and BC populations (Tables S4, S6). The mean number of E-QTL and QQE was 19 with 3.02% of mean PV in RIL population while it was 13 with 3.50% of mean PV in BC population (Table 5). The total number of QTL in both types (62 M-QTLs and 97 E-QTLs) was larger in RIL population than the number of QTL (38 M-QTLs and 67 E-QTL) in BC population. To our surprise, E-QTLs contributed 3.48 and 3.85% of average PV for four traits except fiber uniformity in RIL and BC populations, respectively; whereas M-QTLs explained average PV of 2.41 and 2.42% in RIL and BC populations, respectively. At the same time, QTLs (M-QTLs and E-QTLs) explained 3.04% of PV for four traits on average in both populations whereas environment explained 0.47% of PV on average (Table 5). On the contrary, fiber uniformity displayed vulnerable to environment with 2.89% of average PV.

**Table 5:** Summary of M-QTLs and E-QTLs by environment interaction controlling fiber quality traits by inclusive composite interval mapping in RIL and maternal BC population.

**Trait**	**RIL**			**BC**		
**M-QTLs^a^**	***n*^c^**	**P_(Q)_ (%)^d^**	**P_(QE)_ (%)**	***n***	**P_(Q)_ (%)**	**P_(QE)_ (%)**
Fiber length	13	2.42	0.35	10	2.44	0.57
Fiber uniformity	4	1.67	1.83	3	2.65	4.75
Fiber strength	14	2.86	0.32	9	2.6	0.39
Fiber elongation	21	2.14	0.46	3	2.37	0.99
Micronaire	10	2.23	0.36	13	2.26	0.39
Mean	–	2.26	0.66	–	2.46	1.42
**E-QTLs**^**b**^	***n***	**P_(QQ)_ (%)**	**P_(QQE)_ (%)**	***n***	**P_(QQ)_ (%)**	**P_(QQE)_ (%)**
Fiber length	39	3.37	0.23	10	3.62	0.46
Fiber uniformity	1	1.18	2.92	15	2.1	2.07
Fiber strength	30	3.51	0.28	17	3.46	0.53
Fiber elongation	14	3.53	0.25	3	5.03	0.14
Micronaire	13	3.5	0.84	22	3.3	0.9
Mean	–	3.02	0.90	–	3.50	0.82

a *The single-locus QTLs under multiple environments*.

b *The QTLs of epistasis under multiple environments*.

c *The number of QTLs identified*.

d *P_(Q)_, P_(QE)_, P_(QQ)_, P_(QQE)_ were the mean proportions of total trait phenotypic variances, explained by M-QTL (Q), interaction between M-QTL and environment (QE), epistasis interaction (QQ), and interaction between epistasis and environment (QQE), respectively*.

Here, 18 and seven main-effect QTLs (M-QTLs and QEs) participated in epistatic interactions (E-QTLs and QQE) in RIL and BC populations, respectively (Table 6, Tables S3–S6). Three types of epistasis were checked in RIL population: Type I, both loci were M-QTLs; Type II, either locus between two loci was M-QTL; Type III, both loci were no M-QTLs (Shang et al., 2016d). However, no epistatic QTL with Type I, 7 epistatic QTLs with Type II and 60 epistatic QTLs with Type III were observed in BC population (Table 6). Apparently, Type III was the most popular type of epistatic QTLs in both populations. These results indicated that the epistasis played vital role in improving fiber quality both in RIL and BC populations of Upland cotton. In previous study, epistatic QTLs with significant additive × additive effects were identified for fiber quality traits (Wang B. et al., 2017).

**Table 6:** Types of epistasis detected for fiber quality traits in RIL and BC populations.

	**Type of epistasis^a^**					
**Trait**	**RIL**			**BC**		
	**I**	**II**	**III**	**I**	**II**	**III**
Fiber length	0	3	36	0	2	8
Fiber uniformity	0	1	0	0	1	14
Fiber strength	0	6	24	0	2	15
Fiber elongation	2	4	8	0	0	3
Micronaire	0	2	11	0	2	20
Total	2	16	79	0	7	60

a *Type I, both loci were M-QTLs, Type II, either locus among two loci was M-QTL, and Type III, both loci were no M-QTLs*.

## Discussion

For fiber quality traits, fiber length (FL), fiber strength (FS), and fiber elongation (FE) displayed higher heritability over 0.90 than 0.78 for fiber uniformity (FU) and 0.84 for Micronaire (FM) in RIL population (Table 2). Based on present research and previous results (Shang et al., 2015, 2016c), the performance of “original” maternal parent “GX1135” was superior to those of “original” male parent “GX100-2” for FL, FS, and FE, which was accordance in the result of present study. Taken together, the experiment design was totally finished in six environments across 2 years and four locations (Table S7). Same competition control hybrid (CK) displayed 29.67 and 31.18 mm in 2012E2 and 2015E2, respectively, while “Xinza 1” showed 26.41 and 30.88 mm in 2012E2 and 2015E2, respectively. In addition, 26.96 and 26.53 mm of mean FL values were observed in BC and RIL populations in 2012E2, respectively, while they were 31.01 and 30.85 mm in 2015E2. The results indicated the data validity in present study because of similar variation in “Xinza 1,” the same competition control hybrid and both populations after encountering hailstone disaster in 2015E2.

The identification of stable QTLs across multiple environments and populations plays an essential role in marker-assisted selection (MAS) (Jamshed et al., 2016). In present study, 10 stable QTLs were simultaneously identified in more than one environment or population, of which six stable QTLs focused on Chr 5 (Table 4). Eight QTLs verified each other in the RIL population and its BC progenies as we mentioned above. The results suggested that it is reasonable and effective to map QTLs using the RIL population together with its corresponding BC population across multiple environments and multiple years, which trials were executed in a total of six environments (in present study: 2015E1, 2015E2, 2015E3 and in previous study: 2012E1, 2012E2, 2012E4) by the experiment design using RIL population and its BC population (Shang et al., 2016d).

Twenty QTLs (36.36%) even including seven (70.00%) stable QTLs identified in 2015 were validated the previous result in three environments in 2012 (Shang et al., 2016a; see Table 4, Table S2). Ten of the 20 QTLs were verified each other in two populations. The QTL regions were located on 8 chromosomes. Then, *qFL-Chr5-1* was detected simultaneously across five environments and *qFM-Chr26-1* across four environments including three environments in 2012 in previous study (Shang et al., 2016a). Two QTLs (*qFE-Chr5-1, qFM-Chr9-1*) explained 12.86 and 13.83% of phenotypic variations on average, respectively. In 2008 and 2009, three QTLs near MUSS193 or Gh388 markers co-locating on Chr 5, were also detected across multiple environments using F_2:3_, F_2:4_ segregating populations derived from the hybrid “Xinza 1” (Liang et al., 2013). The region was same to one QTL (*qFL-Chr5-4*) in present study, which closely linked with three other QTLs flanking with HAU1603 and SWU17781. Sun et al. (2012) found one cluster underlying MUSS193a harbored four QTLs in RIL population of Upland cotton. The cluster influenced fiber length, fiber elongation, fiber uniformity, and Micronaire. Markers of NAU6240, PGML1671, PGML1917, SWU17715, Gh388 (SWU17717, NAU4034), and SWU17713 (TMB1296) on Chr 5 across multiple environments, multiple populations, and multiple years will be recommended in MAS to improve fiber quality in Upland cotton. Markers flanking with these stable QTLs detected in multiple populations in present study are valuable for future breeding program and facilitate fine mapping, gene cloning, and favorable gene pyramiding project (Shao et al., 2014).

A total of 251 stable mapped QTLs for fiber quality were detected in the past decade in intraspecific populations derived from Upland cotton (Table S8). Due to the different parents and different genetic maps, few common markers and little common QTL for fiber quality traits in the present BCF_1_ population were found comparing with the previous researches in other labs in Table S8. The *qFU-Chr26-2* controlling fiber uniformity in present study shared the common marker BNL2495 underlying *qFM12.1* controlling Micronaire in previous study (Zhang et al., 2012). Nine markers flanking QTLs for fiber quality traits in Tang's work were common in our map, but no common QTL was observed in comparison with present study (Tang et al., 2015). The *qFS-Chr21-2* explaining 9.06% of PV for fiber strength in present study shared a common SSR marker BNL3171 flanking a stable QTL of *qUHM-21-1* controlling fiber length in BC_3_F_2_ and BC_3_F_2:4_ generation in previous study (Wang B. et al., 2017). Another QTL *qFS-Chr1-1* controlling fiber strength in present study also shared SWU10954 which co-segregated with NAU4045 flanking a stable *qUHM-15-1* controlling fiber length in previous study (Wang B. et al., 2017). In addition, some QTLs in present study located on same chromosomes in accordance with previous studies, such as 13 QTLs locating on Chr 5 in present study, which offered clues and new information for further exploitation. Yang et al. (2015) detected two clusters on Chr 5 and two QTLs on Chr 5 related to fiber uniformity presented in two environments; Zhang et al. (2005) detected one cluster on Chr 5 controlling *LP1* for lint percentage, *FS2* for fiber strength, and *FE1* for fiber elongation. Wang et al. (2016) identified 6 QTLs including two stable QTLs for fiber length distributing on Chr 2, Chr 5, Chr 10, and Chr 19, which were in the shared linkages / chromosomes comparing with present study. Said et al. (2013) summarized 35 QTLs for fiber quality traits (and six QTLs for yield and its components traits) distributed on Chr 5, and 50 QTLs related fiber quality traits distributed on Chr 14. Wang B. et al. (2017) identified the co-location region of BNL2656 and NAU3498 on Chr 5 containing *qUHM-5-1* and *qUI-5-1*, which improved fiber length and fiber uniformity at the same time. In present study, 11 in 12 QTLs on Chr 5 improved fiber length, fiber uniformity, fiber strength, and fiber elongation (Table S2). Further researches need to be performed to interpret the relationship of fiber related genes with stable QTLs in Upland cotton. Recently, the cotton fiber homolog *GhCFE5* on Chr A01 and Chr D05 was found by mapping *GhCFE5A* and *GhCFE5D* in BC_1_ interspecific mapping population, as the same chromosomes in present study (Lv et al., 2016).

The genetic effects were explored in RIL population and its maternal BC progenies. Judging from the overall phenotypic level, RI lines with wider range and maximum values were not obviously worse than some BCF_1S_, whereas a number of lines in RIL population have better phenotypes than that in BC population (Table 1, Table S9). At the single-locus level, all of six QTLs detected in BC population increased 0.24 mm fiber length on average (Table 4, Table S2). It indicated that the heterozygotes increased fiber length a little than the respective homozygotes (Xiao et al., 1995). For fiber length, *qFL-Chr5-3* on Chr 5 increased 0.25 mm in BC population and 0.36 mm in RIL population in 2015E2. At the same time, the QTL increased 0.19 mm in BC population and 0.36 mm in RIL population in 2012E1. The result indicated that the genetic value contributing by one common QTL decreased in BC population in comparison with the value in RIL population.

The epistatic effects and environmental interactions existed commonly for fiber quality traits. Fiber quality traits were sensitive to environment effect (Shao et al., 2014). At two-locus level, there were a number of interactions under environments for fiber quality traits in both populations, but epistasis influenced the performance stronger on average in the maternal BC population than that in RIL population (Table 5). Three types of epistasis combinations were observed (Table 6). Totally, 91 and 81% epistatic interaction of Type III accounted in RIL population and in BC_1_ population, respectively. The result indicated that the Type III was the common epistasis interaction type in both populations. Another interesting result detected that epistasis is another vital genetic effect affecting fiber quality traits following the previous study (Shang et al., 2016d). Similarly to the previous study, Wang et al. (2006) indicated that both epistasis effect and single-locus effect of QTLs played important genetic roles in cotton fiber quality.

There was no evidence that fiber quality traits had heterosis in “Xinza 1” (Table 1). We also found that fiber quality of BCF_1S_ was not apparently superior to its parents in BC population (Table S1). The maximum of fiber length was 34.05 mm in 2015E3 in RIL population but 33.20 mm in BC population, in agreement with that heterozygotes were not always advantageous for performance (Xiao et al., 1995; Hua et al., 2002). For many crops, such as rice and other cereal crops, yield heterosis was obvious so that people have been interested to exploit and interpret the mechanism of hybrid vigor. Differing from these crops, the fiber quality traits of cotton should have different genetic mechanisms in development stage. In nature, plant species needed to produce more seeds for survival in evolution. Obviously, fiber is not necessary for optimal survival. Maybe this is one of the reasons why negative correlation exists between yield and fiber quality (Yang et al., 2015). Further research will be deduced on both heterosis and development of fiber in Upland cotton in order to improve fiber quality and to ultimate heterosis in breeding program.

## Author contributions

LM, YZ, and LS performed the experiments, analyzed the data, and prepared the manuscript. YW maintained the experimental platform and attended bench work. JH conceived the experiments, provided experimental platform, and revised the manuscript.

### Conflict of interest statement

The authors declare that the research was conducted in the absence of any commercial or financial relationships that could be construed as a potential conflict of interest.

## Abbreviations

BC: Backcross population
Chr: Chromosome
CIM: Composite interval mapping
FE: Fiber elongation
FL: Fiber length
FM: Fiber micronaire
FS: Fiber strength
FU: Fiber uniformity
ICIM: Inclusive composite interval mapping
LOD: Likelihood of odd score
QTL: Quantitative trait loci
PV(E): Phenotypic variance (explained)
RIL: Recombinant inbred line population.
