# Supplementary material for: QTLs Analysis and Validation for Fiber Quality Traits Using Maternal Backcross Population in Upland Cotton
Source: Front Plant Sci. 2017 Dec 22;8:2168. doi: 10.3389/fpls.2017.02168 (PMC5744017; doi:10.3389/fpls.2017.02168)
Supplement: Supplementary file 1 [file Table1.DOC]

**TABLE S1 | Summary statistics of MPH for fiber quality traits in BC population** in three environments

| **Trait** | **Env.^1^** | **Mean** | | **SD ^2^** | **Min** | **Max** | **CV%^3^** | **Skewness** | **Kurtosis** |
| --- | --- | --- | --- | --- | --- | --- | --- | --- | --- |
| Fiber length | E1 | | 0.85 | 3.78 | -11.56 | 11.68 | 4.46 | 0.12 | -0.24 |
|  | E2 | | 1.31 | 3.07 | -12.12 | 9.59 | 2.35 | 1.12 | -0.29 |
|  | E3 | | 0.83 | 3.35 | -8.92 | 9.81 | 4.03 | -0.05 | 0.01 |
| Fiber uniformity | E1 | | 1.17 | 6.46 | -19.12 | 22.66 | 5.51 | 0.33 | 0.06 |
|  | E2 | | 0.91 | 4.57 | -11.23 | 18.97 | 5.00 | 0.35 | 0.26 |
|  | E3 | | 0.60 | 5.19 | -14.99 | 19.38 | 8.62 | 0.47 | 0.30 |
| Fiber strength | E1 | | 0.37 | 1.68 | -5.29 | 4.96 | 4.52 | 0.11 | -0.09 |
|  | E2 | | 0.19 | 1.21 | -4.05 | 3.15 | 6.34 | 0.51 | -0.48 |
|  | E3 | | 0.08 | 1.24 | -4.51 | 3.01 | 16.49 | 0.03 | -0.23 |
| Fiber elongation | E1 | | 0.31 | 1.73 | -5.04 | 5.11 | 5.64 | 0.15 | -0.17 |
|  | E2 | | 0.36 | 1.31 | -3.55 | 4.35 | 3.61 | -0.10 | 0.10 |
|  | E3 | | 0.31 | 1.63 | -4.90 | 4.41 | 5.31 | 0.18 | -0.12 |
| Micronaire | E1 | | 2.91 | 10.18 | -33.98 | 42.86 | 3.49 | 1.30 | 0.13 |
|  | E2 | | 0.66 | 7.12 | -18.52 | 23.08 | 10.72 | 0.18 | 0.16 |
|  | E3 | | 1.60 | 4.64 | -17.76 | 26.67 | 2.89 | 4.20 | 0.49 |

*^1^ Environment in 2015, E1, Handan, E2, Cangzhou, E3, Wuhan. ^2^ Standard deviation. ^3^ Coefficient of variation.*
