# Supplementary material for: QTLs Analysis and Validation for Fiber Quality Traits Using Maternal Backcross Population in Upland Cotton
Source: Front Plant Sci. 2017 Dec 22;8:2168. doi: 10.3389/fpls.2017.02168 (PMC5744017; doi:10.3389/fpls.2017.02168)
Supplement: Supplementary file 2 [file Table2.DOC]

**TABLE S2 | Common QTLs for fiber quality traits detected by single locus analysis both in this study and in the previous results (Shang et al., 2016a)**

| **Trait** | **QTL** | **Population** | **Env.^1^** | **Chr ^2^** | **Flanking markers** | | **LOD** | **Effect value^3^** | **Var%^4^** |  |
| --- | --- | --- | --- | --- | --- | --- | --- | --- | --- | --- |
| Fiber length | *qFL-Chr2-1* | RIL | 2015E3 | 2 | DPL0217 | SWU12025 | 3.00 | 0.26 | 5.65 |  |
|  |  | RIL | 2012E2 | 2 | SWU12025 | SWU11889 | 2.13 | 0.62 | 7.92 |  |
|  | ***qFL-Chr5-1***^†^ | RIL | 2015E2 | 5 | SWU20917 | NAU6240 | 2.72 | 0.30 | 7.47 |  |
|  |  | BC | 2015E2 | 5 | NAU6240 | PGML1671 | 2.83 | 0.24 | 10.35 |  |
|  |  | RIL | 2015E3 | 5 | NAU6240 | PGML1671 | 4.63 | 0.35 | 10.11 |  |
|  |  | RIL | 2012E1 | 5 | NAU6240 | PGML1671 | 2.91 | 0.27 | 7.70 |  |
|  |  | RIL | 2012E2 | 5 | SWU20913 | Gh260 | 2.27 | 0.29 | 5.89 |  |
|  |  | BC | 2012E4 | 5 | PGML1671 | PGML1917 | 3.74 | 0.16 | 7.20 |  |
|  | ***qFL-Chr5-2*** | RIL | 2015E1 | 5 | PGML1917 | SWU17715 | 6.83 | 0.61 | 34.00 |  |
|  |  | BC | 2015E2 | 5 | PGML1917 | SWU17715 | 4.78 | 0.28 | 14.07 |  |
|  |  | BC | 2012E1 | 5 | PGML1917 | SWU17715 | 3.12 | 0.29 | 21.09 |  |
|  | ***qFL-Chr5-3*** | RIL | 2015E1 | 5 | Gh388 | SWU17713 | 10.22 | 0.47 | 19.63 |  |
|  |  | BC | 2015E2 | 5 | SWU17713 | HAU1603 | 5.62 | 0.25 | 11.52 |  |
|  |  | RIL | 2015E2 | 5 | Gh388 | SWU17713 | 5.39 | 0.36 | 10.65 |  |
|  |  | BC | 2012E1 | 5 | NAU4034 | SWU17713 | 4.30 | 0.19 | 8.57 |  |
|  |  | RIL | 2012E1 | 5 | NAU4034 | SWU17713 | 8.94 | 0.37 | 16.39 |  |
|  |  | RIL | 2012E1 | 5 | NAU4034 | SWU17713 | 6.39 | 0.34 | 11.68 |  |
|  |  | RIL | 2012E2 | 5 | NAU4034 | SWU17713 | 2.90 | 0.29 | 5.64 |  |
|  | ***qFL-Chr5-4*** | BC | 2015E2 | 5 | PGML4350 | SWU17781 | 3.89 | 0.22 | 8.99 |  |
|  |  | RIL | 2015E3 | 5 | MUSS193 | PGML4350 | 4.44 | 0.32 | 8.55 |  |
|  |  | BC | 2012E4 | 5 | PGML4457 | MUSS193 | 3.15 | 0.15 | 6.24 |  |
|  |  | RIL | 2012E4 | 5 | PGML4457 | MUSS193 | 4.40 | 0.27 | 8.97 |  |
|  |  | RIL | 2012E4 | 5 | PGML4457 | MUSS193 | 4.18 | 0.25 | 7.92 |  |
|  | *qFL-Chr5-5* | RIL | 2015E3 | 5 | NBRI0694 | DPL0022 | 3.66 | 0.29 | 7.13 |  |
|  |  | RIL | 2012E4 | 5 | NBRI0694 | DPL0022 | 4.83 | 0.27 | 9.47 |  |
|  |  | RIL | 2012E4 | 5 | CGR5025 | NBRI0694 | 9.13 | 0.35 | 15.75 |  |
|  |  | BC | 2012E4 | 5 | CGR5025 | NBRI0694 | 4.72 | 0.18 | 9.11 |  |
|  | *qFL-Chr10-1* | RIL | 2015E1 | 10 | SWU20260 | Gh144 | 3.15 | -0.25 | 5.61 |  |
|  |  | RIL | 2012E1 | 10 | ICR00093 | ICR07050 | 3.66 | -0.26 | 8.74 |  |
|  |  | RIL | 2012E2 | 10 | SWU20260 | Gh144 | 3.89 | -0.33 | 7.64 |  |
|  | *qFL-Chr10-2* | RIL | 2015E1 | 10 | Gh320 | HAU0635 | 3.60 | -0.33 | 10.29 |  |
|  |  | RIL | 2012E1 | 10 | Gh320 | HAU0635 | 5.45 | -0.33 | 13.47 |  |
|  |  | RIL | 2012E1 | 10 | Gh320 | HAU0635 | 2.46 | -0.22 | 5.31 |  |
|  | *qFL-Chr19-1* | BC | 2015E2 | 19 | PGML4342 | SWU14431b | 2.76 | 0.25 | 11.16 |  |
|  |  | RIL | 2012E1 | 19 | SWU14431b | SWU17782 | 2.89 | 0.28 | 8.68 |  |
|  |  | BC | 2012E4 | 19 | SWU17782 | DPL0056 | 2.10 | 0.14 | 5.56 |  |
|  |  | RIL | 2012E4 | 19 | SWU14431b | SWU17782 | 4.36 | 0.27 | 9.01 |  |
| Fiber strength | *qFS-Chr5-1* | RIL | 2015E2 | 5 | SWU20917 | NAU6240 | 3.19 | 0.43 | 8.85 |  |
|  |  | RIL | 2012E1 | 5 | NAU6240 | PGML1671 | 5.08 | 0.48 | 11.07 |  |
|  |  | RIL | 2012E2 | 5 | SWU20917 | NAU6240 | 4.73 | 0.43 | 12.58 |  |
|  |  | RIL | 2012E4 | 5 | SWU20917 | NAU6240 | 4.91 | 0.34 | 11.57 |  |
|  |  | RIL | 2012E4 | 5 | SWU20917 | NAU6240 | 6.03 | 0.38 | 14.08 |  |
|  | ***qFS-Chr5-2*** | RIL | 2015E1 | 5 | PGML1917 | SWU17715 | 4.79 | 0.92 | 29.65 |  |
|  |  | BC | 2015E2 | 5 | SWU17715 | Gh388 | 2.85 | 0.26 | 6.00 |  |
|  |  | RIL | 2015E2 | 5 | Gh388 | SWU17713 | 2.72 | 0.33 | 5.13 |  |
|  |  | RIL | 2012E1 | 5 | NAU4034 | SWU17713 | 5.17 | 0.40 | 9.40 |  |
|  |  | BC | 2012E1 | 5 | NAU4034 | SWU17713 | 2.22 | 0.21 | 4.41 |  |
|  | *qFS-Chr21-1* | RIL | 2015E2 | 21 | SWU15915 | SWU0189 | 3.28 | -0.37 | 6.35 |  |
|  |  | RIL | 2015E2 | 21 | SWU0189 | DPL0050a | 2.76 | -0.36 | 6.20 |  |
|  |  | RIL | 2012E1 | 21 | SWU15915 | SWU0189 | 3.20 | -0.32 | 5.87 |  |
|  |  | BC | 2012E2 | 21 | SWU0189 | DPL0050a | 2.16 | -0.21 | 4.72 |  |
|  |  | RIL | 2012E2 | 21 | SWU0189 | DPL0050a | 6.65 | -0.53 | 15.51 |  |
|  | *qFS-Chr21-2* | RIL | 2015E3 | 21 | BNL3171 | CGR5808 | 4.44 | -0.51 | 9.65 |  |
|  |  | RIL | 2012E1 | 21 | BNL3171 | CGR5808 | 3.98 | -0.36 | 7.10 |  |
|  |  | BC | 2012E1 | 21 | BNL3171 | CGR5808 | 2.89 | -0.24 | 5.80 |  |
|  |  | RIL | 2012E2 | 21 | BNL3171 | CGR5808 | 4.52 | -0.43 | 10.60 |  |
| Fiber elongation | *qFE-Chr5-1* | RIL | 2015E1 | 5 | Gh388 | SWU17713 | 7.17 | 0.04 | 14.23 |  |
|  |  | RIL | 2012E1 | 5 | NAU4034 | SWU17713 | 5.40 | 0.06 | 10.35 |  |
|  |  | BC | 2012E1 | 5 | NAU4034 | SWU17713 | 5.26 | 0.04 | 10.77 |  |
|  |  | RIL | 2012E1 | 5 | NAU4034 | SWU17713 | 10.61 | 0.07 | 20.41 |  |
|  |  | RIL | 2012E4 | 5 | NAU4034 | SWU17713 | 4.13 | 0.03 | 8.56 |  |
|  | ***qFE-Chr5-2*** | RIL | 2015E1 | 5 | PGML4457 | MUSS193 | 5.37 | 0.04 | 11.43 |  |
|  |  | RIL | 2015E3 | 5 | HAU1603 | PGML4457 | 3.72 | 0.03 | 8.77 |  |
|  |  | BC | 2012E1 | 5 | PGML4350 | SWU17781 | 3.04 | 0.03 | 6.59 |  |
| Micronaire | *qFM-Chr4-1* | RIL | 2015E2 | 4 | NAU3868 | SWU21617 | 2.76 | 0.09 | 5.67 |  |
|  |  | RIL | 2012E1 | 4 | SWU16783 | NAU3868 | 2.57 | 0.11 | 9.84 |  |
|  |  | RIL | 2012E4 | 4 | NAU3868 | SWU21617 | 2.13 | 0.07 | 3.76 |  |
|  | *qFM-Chr9-1* | RIL | 2015E3 | 9 | NAU2873 | NAU1282 | 2.77 | 0.07 | 5.70 |  |
|  |  | RIL | 2012E4 | 9 | SWU15194 | HAU190 | 4.59 | 0.18 | 25.93 |  |
|  |  | RIL | 2012E4 | 9 | SWU15194 | HAU190 | 3.14 | 0.10 | 9.86 |  |
|  | ***qFM-Chr14-1*** | BC | 2015E1 | 14 | ICR12037 | CGR5675 | 3.59 | 0.11 | 11.81 |  |
|  |  | RIL | 2015E3 | 14 | ICR12037 | CGR5675 | 2.57 | 0.09 | 9.25 |  |
|  |  | RIL | 2012E4 | 14 | ICR12037 | CGR5675 | 3.20 | 0.10 | 8.08 |  |
|  | ***qFM-Chr14-2*** | RIL | 2015E2 | 14 | PGML1368 | PGML1568 | 3.14 | 0.09 | 6.67 |  |
|  |  | RIL | 2015E3 | 14 | PGML1568 | Gh529 | 3.20 | 0.09 | 10.38 |  |
|  |  | RIL | 2012E4 | 14 | PGML1368 | PGML1568 | 3.05 | 0.09 | 6.04 |  |
|  | *qFM-Chr26-1* | BC | 2015E3 | 26 | DPL0070 | NAU2175 | 2.64 | -0.04 | 5.94 |  |
|  |  | RIL | 2012E1 | 26 | SWU17432 | SWU17395 | 2.32 | -0.08 | 5.22 |  |
|  |  | BC | 2012E1 | 26 | NAU2175 | SWU17336 | 4.42 | -0.07 | 9.90 |  |
|  |  | RIL | 2012E2 | 26 | NAU2175 | SWU17336 | 3.97 | -0.09 | 8.00 |  |
|  |  | RIL | 2012E4 | 26 | SWU17432 | SWU17395 | 2.02 | -0.07 | 4.01 |  |

*The single locus QTLs were detected by the software QTL Cartographer (version 2.5).*

*^1^Environment, 2015E1,Handan in 2015, 2015E2, Cangzhou in 2015, 2015E3, Wuhan in 2015, 2012E1, Handan in 2012, 2012E2, Cangzhou in 2012, 2012E4, Xiangyang in 2012.*

*^2^The linkage group number of the loci being tested.*

*^3^The genetic value of a detected QTL, which is the additive effect estimated from the RILs mean values, the additive and dominance effects from maternal BC mean values.*

*^4^Phenotypic variation explained.*

*^†^Bold figures referred to the common QTL detected in this study. Hereinafter same*
