# Supplementary material for: QTLs Analysis and Validation for Fiber Quality Traits Using Maternal Backcross Population in Upland Cotton
Source: Front Plant Sci. 2017 Dec 22;8:2168. doi: 10.3389/fpls.2017.02168 (PMC5744017; doi:10.3389/fpls.2017.02168)
Supplement: Supplementary file 3 [file Table3.DOC]

**TABLE S3 | Main effect QTLs and environmental interactions detected for fiber quality traits in RIL populations using two-locus analysis**

| **Trait** | **Chr** | **Position  (cM)** | **Flanking markers** | | **LOD** | **V(A)%^1^** | **V(AE)%** | **Effecct value^2^** | | | |
| --- | --- | --- | --- | --- | --- | --- | --- | --- | --- | --- | --- |
|  |  |  |  | |  |  |  | **A** | **AE1** | **AE2** | **AE3** |
| FL | 1 | 10 | SWU10912 | DPL0090 | 2.81 | 1.79 | 0.15 | 0.14 | -0.06 | 0.02 | 0.04 |
|  | 2 | 4 | **SWU12147**^†^ | **CGR6695** | 7.52 | 4.94 | 0.05 | 0.24 | 0.03 | -0.03 | -0.01 |
|  | 2 | 65 | SWU11950 | TMB1268 | 4.12 | 2.29 | 0.65 | 0.16 | -0.03 | 0.12 | -0.09 |
|  | 5 | 20 | **SWU20917** | **NAU6240** | 4.01 | 2.36 | 0.35 | 0.16 | -0.04 | 0.09 | -0.05 |
|  | 5 | 52 | **NAU6240** | **PGML1671** | 4.46 | 2.52 | 0.60 | 0.17 | -0.02 | -0.09 | 0.11 |
|  | 5 | 123 | **MUSS193** | **PGML4350** | 4.30 | 1.82 | 1.56 | 0.14 | -0.11 | -0.07 | 0.19 |
|  | 7 | 143 | SWU10785 | CER0036 | 3.00 | 1.69 | 0.44 | -0.14 | 0.09 | 0.00 | -0.09 |
|  | 10 | 42 | SWU20260 | Gh144 | 5.50 | 3.40 | 0.14 | -0.20 | -0.06 | 0.03 | 0.02 |
|  | 13 | 79 | BNL1495 | CGR5390 | 4.04 | 2.70 | 0.05 | 0.18 | -0.03 | 0.02 | 0.01 |
|  | 14 | 123 | PGML1568 | Gh529 | 2.82 | 1.77 | 0.12 | -0.14 | 0.01 | 0.04 | -0.05 |
|  | 19 | 119 | **PGML4342** | **SWU14431b** | 2.72 | 1.65 | 0.21 | 0.14 | -0.01 | -0.05 | 0.06 |
|  | 21 | 156 | SWU14431a | SWU15915 | 3.51 | 2.31 | 0.10 | -0.16 | 0.05 | -0.02 | -0.02 |
|  | 21 | 167 | SWU0189 | DPL0050a | 3.58 | 2.26 | 0.24 | -0.16 | 0.07 | -0.03 | -0.05 |
| FU | 1 | 155 | NAU3384 | CGR5663 | 2.55 | 1.81 | 0.03 | 0.12 | 0.01 | 0.01 | -0.02 |
|  | 2 | 3 | SWU12126 | SWU12147 | 2.66 | 0.13 | 2.07 | 0.03 | 0.18 | -0.08 | -0.10 |
|  | 5 | 114 | SWU17715 | Gh388 | 7.80 | 4.53 | 2.76 | 0.19 | 0.21 | -0.10 | -0.11 |
|  | 5 | 152 | SWU13378 | SWU17846 | 2.99 | 0.21 | 2.45 | -0.04 | -0.20 | 0.09 | 0.11 |
| FS | 2 | 6 | DPL0217 | SWU12025 | 2.85 | 1.92 | 0.07 | 0.22 | 0.05 | 0.00 | -0.05 |
|  | 2 | 65 | SWU11950 | TMB1268 | 5.83 | 3.69 | 0.24 | 0.31 | -0.04 | 0.11 | -0.07 |
|  | 5 | 25 | SWU20917 | NAU6240 | 6.50 | 4.22 | 0.17 | 0.33 | -0.08 | -0.01 | 0.08 |
|  | 5 | 114 | **SWU17715** | **Gh388** | 8.64 | 5.95 | 0.56 | 0.39 | 0.16 | -0.02 | -0.13 |
|  | 7 | 38 | NAU3181 | SHIN0376 | 3.20 | 1.95 | 0.40 | 0.22 | 0.07 | -0.14 | 0.07 |
|  | 8 | 31 | Gh197 | DC20094 | 3.33 | 2.18 | 0.01 | 0.23 | -0.01 | 0.03 | -0.02 |
|  | 13 | 84 | BNL1495 | CGR5390 | 3.72 | 2.45 | 0.43 | 0.25 | 0.15 | -0.07 | -0.08 |
|  | 14 | 99 | PGML1368 | PGML1568 | 3.34 | 2.32 | 0.02 | -0.24 | -0.02 | 0.03 | -0.02 |
|  | 15 | 4 | DC40175 | SWU11630 | 3.27 | 2.00 | 0.30 | 0.23 | -0.08 | -0.05 | 0.12 |
|  | 18 | 77 | SWU21800 | CIR099 | 4.26 | 2.94 | 0.10 | 0.27 | 0.07 | -0.04 | -0.03 |
|  | 20 | 6 | CGR5548 | SWU20675 | 3.80 | 2.39 | 0.13 | -0.25 | 0.08 | -0.01 | -0.06 |
|  | 21 | 157 | SWU15915 | SWU0189 | 4.67 | 2.37 | 0.62 | -0.25 | 0.00 | -0.16 | 0.15 |
|  | 21 | 175 | **CGR5808** | **HAU0423** | 6.08 | 3.42 | 1.36 | -0.29 | -0.01 | 0.23 | -0.22 |
|  | 24 | 90 | SWU13758 | CGR5423 | 3.27 | 2.21 | 0.13 | -0.24 | -0.08 | 0.05 | 0.04 |
| FE | 1 | 21 | DPL0090 | Gh398 | 4.62 | 1.65 | 0.53 | 0.01 | -0.01 | 0.01 | 0.00 |
|  | 2 | 2 | SWU12126 | SWU12147 | 2.98 | 1.86 | 0.02 | 0.01 | 0.00 | 0.00 | 0.00 |
|  | 2 | 58 | SWU11976 | SWU11950 | 3.12 | 2.01 | 0.08 | 0.01 | 0.00 | 0.00 | 0.00 |
|  | 2 | 66 | PGML0700 | SWU12016 | 3.53 | 2.13 | 0.09 | 0.02 | 0.00 | 0.00 | 0.00 |
|  | 5 | 1 | SWU20913 | Gh260 | 5.11 | 3.07 | 0.92 | 0.02 | 0.00 | -0.01 | 0.01 |
|  | 5 | 50 | NAU6240 | PGML1671 | 3.15 | 1.93 | 0.13 | 0.01 | 0.00 | 0.00 | 0.00 |
|  | 5 | 114 | **SWU17715** | **Gh388** | 8.03 | 3.84 | 2.68 | 0.02 | 0.02 | -0.01 | -0.02 |
|  | 5 | 119 | HAU1603 | PGML4457 | 4.76 | 2.04 | 1.46 | 0.01 | -0.01 | 0.00 | 0.02 |
|  | 7 | 145 | CER0036 | PGML1916 | 3.57 | 2.22 | 0.61 | -0.02 | 0.00 | 0.01 | -0.01 |
|  | 9 | 151 | **NAU3966** | **SWU15157** | 3.52 | 2.06 | 0.02 | -0.01 | 0.00 | 0.00 | 0.00 |
|  | 10 | 1 | BNL2960 | SWU20511 | 2.60 | 1.68 | 0.13 | -0.01 | 0.00 | 0.00 | 0.00 |
|  | 10 | 62 | Gh320 | HAU0635 | 2.65 | 1.63 | 0.12 | -0.01 | 0.00 | 0.00 | 0.00 |
|  | 11 | 3 | NAU3390 | NAU2460 | 6.36 | 3.70 | 0.03 | -0.02 | 0.00 | 0.00 | 0.00 |
|  | 13 | 81 | BNL1495 | CGR5390 | 4.89 | 3.03 | 0.42 | 0.02 | 0.01 | -0.01 | 0.00 |
|  | 15 | 18 | DPL0182 | SWU11691 | 3.68 | 1.72 | 1.33 | -0.01 | -0.02 | 0.01 | 0.01 |
|  | 16 | 80 | SWU10214 | Gh56 | 3.35 | 2.17 | 0.07 | 0.02 | 0.00 | 0.00 | 0.00 |
|  | 16 | 98 | CGR6802 | HAU1129 | 2.63 | 1.22 | 0.22 | 0.01 | -0.01 | 0.00 | 0.00 |
|  | 16 | 149 | SWU10094 | SWU10060 | 2.89 | 1.75 | 0.01 | 0.01 | 0.00 | 0.00 | 0.00 |
|  | 24 | 33 | Gh268 | SWU13268 | 5.11 | 2.83 | 0.11 | -0.02 | 0.00 | 0.00 | 0.00 |
|  | 25 | 130 | Gh220 | SWU19434 | 2.97 | 0.94 | 0.45 | 0.01 | 0.00 | 0.01 | -0.01 |
|  | 28 | 84 | TMB2386 | SWU12343 | 2.55 | 1.63 | 0.30 | 0.01 | 0.00 | -0.01 | 0.00 |
| FM | 2 | 65 | SWU11950 | TMB1268 | 2.66 | 1.92 | 0.37 | -0.05 | 0.00 | -0.03 | 0.03 |
|  | 4 | 91 | SWU16783 | NAU3868 | 3.01 | 1.83 | 0.50 | 0.05 | -0.03 | 0.03 | -0.01 |
|  | 5 | 14 | **SWU20913** | **Gh260** | 2.92 | 0.67 | 1.29 | -0.03 | 0.05 | -0.02 | -0.04 |
|  | 9 | 59 | SWU15194 | HAU190 | 3.66 | 2.72 | 0.04 | 0.06 | -0.01 | 0.01 | 0.00 |
|  | 9 | 72 | HAU1618 | NAU2873 | 4.24 | 3.30 | 0.06 | 0.06 | 0.01 | -0.01 | 0.00 |
|  | 11 | 0 | NAU3390 | NAU2460 | 3.70 | 2.69 | 0.65 | 0.06 | 0.00 | 0.04 | -0.03 |
|  | 14 | 107 | **PGML1568** | **Gh529** | 4.33 | 3.09 | 0.03 | 0.06 | -0.01 | 0.01 | 0.00 |
|  | 18 | 117 | **NAU748** | **SWU22192** | 2.53 | 1.56 | 0.22 | 0.04 | -0.02 | 0.01 | 0.01 |
|  | 19 | 123 | **SWU14431b** | **SWU17782** | 3.17 | 2.18 | 0.24 | -0.05 | 0.01 | -0.02 | 0.01 |
|  | 19 | 171 | CAU0104 | SWU17897 | 2.92 | 2.35 | 0.19 | -0.05 | -0.01 | -0.01 | 0.02 |

*The result detected by software ICIMapping 4.1.*

*^1^V(A)% and V(AE)%, percentage of the total phenotypic variation explained by one QTL and by QTL × environmentat the current scanning position, respectively.*

*^2^A, the genetic effect of one QTL; AE, the effect values by QTL × environment; AE1, AE2 and AE3: the effect values under environments of E01, E02 and E03, respectively.*

*^†^Bold figures indicated common QTLs between E-QTLs by QEs and M-QTLs by QEs in the population. Hereinafter same*
