# Supplementary material for: QTLs Analysis and Validation for Fiber Quality Traits Using Maternal Backcross Population in Upland Cotton
Source: Front Plant Sci. 2017 Dec 22;8:2168. doi: 10.3389/fpls.2017.02168 (PMC5744017; doi:10.3389/fpls.2017.02168)
Supplement: Supplementary file 4 [file Table4.DOC]

**TABLE S4 | Epistatic effect QTLs and environmental interactions detected for fiber quality traits in RIL population using two-locus analysis**

| **Trait** | **Ch*i*^1^** | **Flanking markers** | | **Ch*j*** | **Flanking markers** | | **LOD** | **V(AA)%^2^** | **V(AAE)%** | **Effect value^3^** | | | |
| --- | --- | --- | --- | --- | --- | --- | --- | --- | --- | --- | --- | --- | --- |
|  |  |  | |  |  | |  |  |  | **AA** | **AAE1** | **AAE2** | **AAE3** |
| FL | 1 | **DPL0090** | **Gh398** | 3 | SWU12840 | NAU2742 | 5.48 | 3.10 | 0.11 | -0.19 | 0.05 | -0.03 | -0.02 |
|  | 2 | PGML0700 | SWU12016 | 5 | TMB1296 | HAU1603 | 7.32 | 4.33 | 0.13 | 0.22 | -0.01 | -0.05 | 0.05 |
|  | 5 | SWU20913 | Gh260 | 7 | CGR5372 | SWU10205 | 6.70 | 3.50 | 0.60 | 0.20 | -0.07 | -0.04 | 0.12 |
|  | 1 | ICR03724 | ICR03725 | 7 | SWU10205 | HAU1483a | 7.00 | 4.32 | 0.05 | -0.22 | 0.04 | 0.00 | -0.03 |
|  | 4 | SWU12672 | HAU1332 | 9 | Gh111 | Gh27 | 5.30 | 3.13 | 0.17 | 0.19 | -0.06 | 0.04 | 0.02 |
|  | 7 | PGML1916 | SWU10864 | 10 | NAU2139 | SWU20689 | 6.28 | 3.57 | 0.08 | -0.21 | -0.01 | 0.04 | -0.03 |
|  | 11 | PGML2202 | CGR6580 | 12 | NAU943 | DPL0303 | 5.78 | 3.27 | 0.21 | 0.19 | -0.07 | 0.04 | 0.03 |
|  | 1 | CGR5663 | NAU2343 | 13 | NAU2893 | Gh157 | 5.45 | 3.27 | 0.01 | 0.20 | 0.00 | 0.01 | -0.01 |
|  | 3 | HAU2424 | CER0028 | 15 | DPL0182 | SWU11691 | 5.84 | 3.62 | 0.02 | -0.20 | 0.02 | -0.03 | 0.01 |
|  | 5 | PGML1917 | SWU17715 | 16 | DPL0048 | SWU10266 | 6.00 | 3.61 | 0.16 | -0.21 | -0.03 | 0.06 | -0.04 |
|  | 1 | SWU10986 | NAU2218 | 18 | CIR099 | NAU748 | 5.96 | 3.05 | 0.57 | 0.19 | -0.11 | 0.08 | 0.03 |
|  | 4 | SWU18876 | SWU12672 | 18 | CIR099 | NAU748 | 6.80 | 3.98 | 0.29 | 0.21 | 0.04 | -0.08 | 0.05 |
|  | 7 | CGR5372 | SWU10205 | 19 | NAU5330 | Gh72 | 6.77 | 4.11 | 0.09 | -0.22 | 0.04 | -0.01 | -0.03 |
|  | 6 | ICR03206 | NAU896 | 19 | DC40122 | NAU833a | 5.51 | 3.25 | 0.11 | 0.20 | -0.02 | 0.05 | -0.03 |
|  | 11 | NAU3390 | NAU2460 | 19 | SWU17782 | DPL0056 | 6.14 | 2.63 | 0.86 | 0.18 | -0.12 | 0.00 | 0.12 |
|  | 17 | SWU14627 | CGR5871 | 20 | CGR6154 | SWU20246 | 5.67 | 3.10 | 0.30 | 0.19 | -0.08 | 0.01 | 0.07 |
|  | 13 | DPL0572 | HAU2558 | 20 | SWU20246 | SWU20501a | 5.16 | 2.77 | 0.21 | 0.18 | -0.07 | 0.05 | 0.02 |
|  | 18 | DC40150 | ICR02849 | 20 | SWU20246 | SWU20501a | 5.19 | 2.91 | 0.19 | 0.18 | -0.07 | 0.04 | 0.03 |
|  | 9 | Gh27 | SWU15194 | 20 | CER0167 | SWU20064 | 7.12 | 3.85 | 0.27 | 0.21 | -0.08 | 0.03 | 0.05 |
|  | 1 | CGR6129 | DPL0790 | 21 | SWU0830 | HAU2004 | 6.91 | 4.16 | 0.07 | 0.22 | 0.01 | -0.04 | 0.02 |
|  | 11 | ICR01810 | CGR6525 | 21 | **SWU14431a** | **SWU15915** | 5.97 | 3.26 | 0.40 | 0.20 | -0.03 | -0.07 | 0.09 |
|  | 12 | Gh631 | HAU1321 | 21 | **SWU0189** | **DPL0050a** | 5.85 | 3.52 | 0.06 | 0.20 | -0.04 | 0.01 | 0.03 |
|  | 1 | NAU3384 | CGR5663 | 23 | PGML4186 | NAU3100 | 5.46 | 3.29 | 0.05 | -0.20 | 0.01 | 0.02 | -0.03 |
|  | 8 | DC20094 | HAU1470b | 23 | PGML4186 | NAU3100 | 6.16 | 3.21 | 0.49 | -0.19 | 0.08 | 0.03 | -0.10 |
|  | 14 | ICR12037 | CGR5675 | 23 | PGML4186 | NAU3100 | 6.82 | 3.95 | 0.11 | 0.21 | 0.05 | -0.05 | 0.00 |
|  | 8 | DC20094 | HAU1470b | 24 | CGR5202 | Gh298 | 5.53 | 3.30 | 0.10 | 0.20 | -0.03 | 0.04 | -0.01 |
|  | 19 | NAU3437 | NAU2894 | 24 | BNL1521 | HAU2504 | 6.70 | 3.55 | 0.41 | 0.21 | -0.09 | 0.08 | 0.01 |
|  | 13 | DPL0894 | SWU10800 | 25 | SWU19815 | BNL3594 | 5.47 | 3.16 | 0.17 | 0.19 | -0.06 | 0.02 | 0.05 |
|  | 16 | NAU2984 | SWU10062 | 26 | BNL2495 | DPL0491 | 5.06 | 2.97 | 0.13 | -0.19 | 0.03 | -0.05 | 0.02 |
|  | 13 | SHIN1462 | SWU22374 | 26 | C2_0135 | PGML2321 | 5.33 | 2.68 | 0.43 | 0.18 | -0.10 | 0.07 | 0.03 |
|  | 24 | BNL1521 | HAU2504 | 26 | C2_0135 | PGML2321 | 5.39 | 3.22 | 0.20 | 0.19 | -0.06 | 0.00 | 0.06 |
|  | 1 | HAU1417 | NAU2437 | 26 | SWU18488 | SWU18672 | 6.34 | 3.75 | 0.13 | 0.21 | -0.05 | 0.05 | 0.00 |
|  | 19 | SWU17882 | CAU0104 | 26 | SWU18681 | SWU0598 | 6.01 | 3.05 | 0.48 | 0.19 | -0.09 | 0.00 | 0.09 |
|  | 21 | BNL3171 | CGR5808 | 27 | SWU10994 | HAU1001 | 5.01 | 2.72 | 0.22 | 0.18 | -0.07 | 0.02 | 0.05 |
|  | 18 | SWU22281 | SWU21800 | 28 | HAU3071 | CGR5534 | 5.22 | 3.12 | 0.08 | 0.19 | 0.04 | 0.00 | -0.03 |
|  | 2 | PGML0700 | SWU12016 | 28 | SHIN0219 | TMB2386 | 7.86 | 3.75 | 0.94 | 0.21 | -0.14 | 0.03 | 0.11 |
|  | 7 | PGML1916 | SWU10864 | 28 | SHIN0219 | TMB2386 | 5.18 | 2.97 | 0.16 | 0.19 | -0.05 | 0.01 | 0.04 |
|  | 11 | NAU1014 | ICR10344 | 28 | SHIN0219 | TMB2386 | 5.07 | 3.16 | 0.00 | -0.19 | 0.00 | -0.01 | 0.00 |
|  | 11 | CGR5421 | ICR08245 | 29 | C2_0115 | ICR03107 | 5.33 | 3.13 | 0.08 | -0.19 | 0.04 | -0.02 | -0.02 |
| FU | 1 | **NAU3384** | **CGR5663** | 14 | PGML1568 | Gh529 | 5.36 | 1.18 | 2.92 | 0.10 | 0.13 | 0.08 | -0.21 |
| FS | 2 | SWU12126 | SWU12147 | 4 | SWU16782 | SWU16783 | 5.83 | 3.48 | 0.17 | -0.30 | -0.08 | 0.08 | 0.00 |
|  | 2 | SWU11887 | SWU11976 | 5 | **NAU6240** | **PGML1671** | 5.23 | 3.00 | 0.02 | 0.27 | 0.03 | -0.03 | 0.00 |
|  | 4 | ICR01729 | SWU16781 | 6 | ICR03206 | NAU896 | 5.27 | 2.41 | 0.73 | 0.25 | 0.00 | -0.16 | 0.17 |
|  | 8 | **CGR6508** | **Gh197** | 8 | DC20094 | HAU1470b | 5.01 | 2.90 | 0.17 | -0.28 | -0.02 | 0.09 | -0.07 |
|  | 6 | ICR00143 | CGR5108 | 13 | SWU22413 | CGR5331 | 6.63 | 3.87 | 0.37 | -0.32 | -0.13 | 0.03 | 0.10 |
|  | 7 | NAU1357 | SWU10067 | 16 | PGML1709 | SWU10627 | 6.97 | 3.06 | 1.49 | -0.29 | -0.28 | 0.14 | 0.13 |
|  | 5 | **SWU20917** | **NAU6240** | 16 | SWU10627 | PGML1309 | 6.22 | 4.00 | 0.06 | -0.32 | 0.00 | -0.05 | 0.05 |
|  | 11 | CGR5421 | ICR08245 | 16 | SWU10214 | Gh56 | 5.02 | 2.88 | 0.20 | -0.27 | 0.10 | -0.06 | -0.04 |
|  | 1 | SWU11191 | BNL2827b | 16 | Gh56 | NAU5120 | 5.51 | 2.84 | 0.62 | -0.27 | 0.11 | 0.07 | -0.18 |
|  | 14 | PGML4763 | SWU13909 | 16 | SWU10038 | ICR00016 | 6.18 | 3.55 | 0.24 | -0.30 | -0.06 | 0.11 | -0.05 |
|  | 16 | CGR6802 | HAU1129 | 16 | SWU10038 | ICR00016 | 5.88 | 3.42 | 0.26 | -0.30 | -0.09 | 0.11 | -0.02 |
|  | 4 | BNL1167 | SWU21415 | 20 | SWU20636 | CGR6154 | 6.23 | 3.90 | 0.19 | 0.32 | 0.07 | -0.10 | 0.02 |
|  | 12 | Gh631 | HAU1321 | 21 | SWU16651 | SWU16645 | 6.56 | 3.92 | 0.10 | -0.32 | -0.06 | 0.06 | 0.00 |
|  | 9 | NAU3966 | SWU15157 | 21 | **SWU14431a** | **SWU15915** | 5.06 | 2.83 | 0.41 | 0.27 | 0.01 | -0.12 | 0.11 |
|  | 8 | **CGR6508** | **Gh197** | 21 | CGR5806 | DPL0777 | 5.34 | 3.02 | 0.06 | -0.28 | 0.04 | -0.04 | 0.00 |
|  | 11 | SWU15972 | TMB0628 | 21 | CGR5217 | BNL3442a | 6.26 | 3.54 | 0.37 | 0.30 | 0.03 | -0.13 | 0.10 |
|  | 1 | NAU2218 | SWU11191 | 21 | CGR5217 | BNL3442a | 6.45 | 3.75 | 0.04 | 0.32 | 0.01 | -0.05 | 0.04 |
|  | 2 | SWU12126 | SWU12147 | 22 | SWU21533 | DPL0562 | 7.50 | 4.50 | 0.28 | -0.35 | 0.07 | 0.05 | -0.12 |
|  | 22 | PGML1712 | SWU21538 | 22 | PGML0695 | SWU20813 | 8.34 | 5.07 | 0.20 | -0.36 | -0.08 | 0.10 | -0.01 |
|  | 1 | NAU3384 | CGR5663 | 23 | PGML4186 | NAU3100 | 7.99 | 4.67 | 0.01 | -0.35 | 0.02 | 0.01 | -0.03 |
|  | 14 | ICR12037 | CGR5675 | 23 | PGML4186 | NAU3100 | 7.09 | 4.28 | 0.11 | 0.33 | 0.08 | -0.03 | -0.05 |
|  | 1 | HAU1417 | NAU2437 | 24 | PGML4657 | Gh454 | 5.62 | 3.42 | 0.12 | -0.30 | -0.02 | 0.07 | -0.05 |
|  | 21 | SWU0830 | HAU2004 | 26 | MGHES31 | HAU1571 | 5.56 | 3.10 | 0.17 | 0.29 | -0.04 | -0.06 | 0.10 |
|  | 9 | SWU15157 | SWU14934 | 26 | BNL2495 | DPL0491 | 5.68 | 3.29 | 0.26 | -0.29 | -0.07 | 0.11 | -0.04 |
|  | 16 | CGR6802 | HAU1129 | 26 | DPL0491 | Gh64 | 5.02 | 2.82 | 0.24 | -0.27 | 0.11 | -0.06 | -0.05 |
|  | 23 | SWU14807 | PGML4185 | 26 | DPL0491 | Gh64 | 5.32 | 2.93 | 0.33 | -0.28 | -0.05 | 0.14 | -0.09 |
|  | 13 | **BNL1495** | **CGR5390** | 29 | DC20127 | DPL0252 | 7.72 | 4.78 | 0.08 | -0.35 | 0.00 | 0.05 | -0.06 |
|  | 25 | DPL0282 | SWU19763 | 29 | BNL3261 | CGR5111 | 5.02 | 2.72 | 0.42 | -0.26 | -0.01 | 0.13 | -0.12 |
|  | 28 | CGR5534 | SHIN0219 | 31 | SWU16676 | SWU16755 | 6.83 | 3.74 | 0.52 | 0.31 | -0.06 | -0.10 | 0.16 |
|  | 11 | NAU3695 | DPL0050b | 32 | NAU2140 | NAU2957 | 5.91 | 3.48 | 0.28 | -0.30 | -0.12 | 0.07 | 0.04 |
| FE | 1 | ICR03724 | ICR03725 | 1 | SWU10986 | NAU2218 | 5.05 | 3.29 | 0.47 | 0.02 | 0.00 | -0.01 | 0.02 |
|  | 1 | NAU2218 | SWU11191 | 5 | **HAU1603** | **PGML4457** | 5.33 | 3.37 | 0.29 | -0.02 | 0.00 | 0.01 | -0.01 |
|  | 9 | SWU15194 | HAU190 | 9 | **SWU15157** | **SWU14934** | 5.11 | 3.27 | 0.22 | -0.02 | 0.01 | 0.00 | -0.01 |
|  | 13 | SHIN1462 | SWU22374 | 13 | HAU2558 | NAU2893 | 5.08 | 2.99 | 0.40 | 0.02 | 0.01 | -0.01 | 0.00 |
|  | 13 | SHIN1462 | SWU22374 | 14 | SWU13909 | TMB0071 | 5.03 | 3.33 | 0.29 | 0.02 | 0.01 | 0.00 | 0.00 |
|  | 6 | ICR00143 | CGR5108 | 14 | BNL3661 | PGML2498 | 5.90 | 3.76 | 0.18 | 0.02 | 0.01 | -0.01 | 0.00 |
|  | 5 | **PGML1671** | **PGML1917** | 16 | **SWU10214** | **Gh56** | 5.26 | 3.45 | 0.18 | -0.02 | 0.00 | 0.01 | 0.00 |
|  | 16 | SWU10038 | ICR00016 | 17 | SWU12818 | CGR5576 | 5.49 | 3.51 | 0.18 | -0.02 | 0.00 | 0.00 | 0.00 |
|  | 6 | ICR10602 | SWU19656 | 20 | SWU20246 | SWU20501a | 5.02 | 2.87 | 0.07 | 0.02 | 0.00 | 0.00 | 0.00 |
|  | 6 | ICR00143 | CGR5108 | 21 | **SWU0830** | **HAU2004** | 6.82 | 4.42 | 0.40 | 0.02 | 0.01 | -0.01 | 0.00 |
|  | 1 | **DPL0090** | **Gh398** | 21 | **SWU0830** | **HAU2004** | 8.26 | 5.43 | 0.18 | 0.02 | 0.00 | -0.01 | 0.00 |
|  | 22 | SWU21533 | DPL0562 | 22 | PGML0695 | SWU20813 | 5.86 | 3.52 | 0.32 | -0.02 | -0.01 | 0.01 | 0.00 |
|  | 2 | PGML0700 | SWU12016 | 25 | BNL3594 | DPL0282 | 5.17 | 3.18 | 0.00 | -0.02 | 0.00 | 0.00 | 0.00 |
|  | 11 | SWU15972 | TMB0628 | 28 | **SHIN0219** | **TMB2386** | 5.04 | 3.10 | 0.36 | -0.02 | -0.01 | 0.01 | 0.00 |
| FM | 2 | **SWU11950** | **TMB1268** | 13 | SHIN1462 | SWU22374 | 5.58 | 3.18 | 0.97 | -0.06 | -0.05 | 0.02 | 0.03 |
|  | 1 | SWU11191 | BNL2827b | 16 | SWU10627 | PGML1309 | 5.13 | 3.67 | 0.23 | 0.07 | 0.02 | 0.00 | -0.02 |
|  | 6 | SWU19541 | CGR5801 | 19 | **SWU17897** | **CGR5539** | 6.48 | 2.24 | 3.12 | -0.05 | -0.08 | 0.06 | 0.02 |
|  | 14 | NAU3820 | NAU2960 | 20 | SWU20700 | CGR5548 | 5.12 | 3.32 | 0.74 | 0.06 | 0.04 | -0.02 | -0.03 |
|  | 10 | SWU20501b | CGR5873 | 21 | SWU14431a | SWU15915 | 5.23 | 3.44 | 0.45 | -0.07 | -0.03 | -0.01 | 0.04 |
|  | 5 | CGR5025 | NBRI0694 | 21 | CGR5808 | HAU0423 | 5.11 | 3.58 | 0.25 | 0.07 | -0.01 | 0.02 | -0.01 |
|  | 11 | NAU1014 | ICR10344 | 21 | CGR5217 | BNL3442a | 5.32 | 4.01 | 0.21 | -0.07 | 0.01 | -0.02 | 0.01 |
|  | 23 | SWU14807 | PGML4185 | 25 | HAU1382 | SWU19848 | 8.00 | 6.13 | 0.21 | 0.09 | 0.02 | -0.01 | -0.01 |
|  | 22 | PGML0695 | SWU20813 | 25 | DPL0282 | SWU19763 | 6.01 | 3.94 | 0.21 | 0.07 | -0.01 | 0.02 | -0.01 |
|  | 26 | BNL598 | PGML1637 | 26 | SWU17395 | DC30107 | 5.17 | 3.14 | 0.90 | 0.07 | 0.06 | -0.03 | -0.03 |
|  | 11 | CER0098 | CGR5421 | 26 | SWU18681 | SWU0598 | 5.10 | 0.89 | 3.15 | 0.03 | 0.09 | -0.05 | -0.03 |
|  | 26 | SWU0514 | SWU18488 | 27 | CGR6857 | ICR11205 | 5.09 | 3.51 | 0.27 | -0.07 | -0.03 | 0.01 | 0.02 |
|  | 4 | SWU18876 | SWU12672 | 28 | CGR5534 | SHIN0219 | 6.16 | 4.39 | 0.15 | 0.07 | 0.01 | 0.00 | -0.02 |

*The result detected by software ICIMapping 4.1.*

*^1^Chi and Chj represented the linkage group number of the loci being tested in the analysis.*

*^2^V(AA) and V(AAE), percentage of the total phenotypic variation, explaining by epistasis QTLs and by epistasis QTLs × the environment, respectively.*

*^3^AA, the total effect value of epistasis QTLs; AAEl, AAE2, and AAE3 indicated the effects by epistatic QTLs × environment in E1, E2 and E3, respectively. Hereinafter same*
