# Supplementary material for: QTLs Analysis and Validation for Fiber Quality Traits Using Maternal Backcross Population in Upland Cotton
Source: Front Plant Sci. 2017 Dec 22;8:2168. doi: 10.3389/fpls.2017.02168 (PMC5744017; doi:10.3389/fpls.2017.02168)
Supplement: Supplementary file 5 [file Table5.DOC]

**TABLE S5 | Main effect QTLs and environmental interactions detected for fiber quality traits in BC population using two-locus analysis**

| **Trait** | **Chr** | **Position (cM)** | **Flanking markers** | | **LOD** | | **V(A)%** | | **V(AE)%** | | **Effect value** | | | | | | |
| --- | --- | --- | --- | --- | --- | --- | --- | --- | --- | --- | --- | --- | --- | --- | --- | --- | --- |
|  |  |  |  | |  | |  | |  | | **A** | | **AE1** | | **AE2** | | **AE3** |
| FL | 2 | 4 | **SWU12147** | **CGR6695** | | 2.92 | | 2.71 | | 0.06 | | 0.11 | | -0.02 | | 0.02 | 0.00 |
|  | 4 | 0 | SWU18881 | NAU2701 | | 3.19 | | 2.12 | | 1.18 | | 0.10 | | -0.06 | | -0.05 | 0.11 |
|  | 5 | 48 | **NAU6240** | **PGML1671** | | 2.96 | | 1.40 | | 0.97 | | 0.08 | | -0.10 | | 0.06 | 0.03 |
|  | 5 | 121 | **PGML4457** | **MUSS193** | | 2.96 | | 2.31 | | 0.88 | | 0.11 | | 0.06 | | -0.09 | 0.04 |
|  | 5 | 131 | NBRI0694 | DPL0022 | | 3.79 | | 3.42 | | 0.50 | | 0.13 | | 0.02 | | -0.07 | 0.04 |
|  | 14 | 58 | NAU3308 | HAU1057 | | 2.92 | | 2.30 | | 1.21 | | -0.11 | | -0.11 | | 0.07 | 0.03 |
|  | 19 | 116 | **PGML4342** | **SWU14431b** | | 3.32 | | 2.87 | | 0.10 | | 0.12 | | -0.01 | | 0.03 | -0.02 |
|  | 19 | 171 | CAU0104 | SWU17897 | | 2.84 | | 2.69 | | 0.14 | | 0.11 | | 0.02 | | 0.01 | -0.04 |
|  | 22 | 2 | SWU21646 | SWU21585 | | 2.73 | | 1.88 | | 0.65 | | 0.10 | | -0.07 | | 0.01 | 0.06 |
|  | 30 | 11 | TMB1638 | CGR6812 | | 2.78 | | 2.73 | | 0.04 | | 0.11 | | -0.01 | | -0.01 | 0.02 |
| FU | 9 | 0 | Gh111 | Gh27 | | 3.24 | | 0.22 | | 6.40 | | 0.02 | | -0.11 | | 0.16 | -0.05 |
|  | 15 | 5 | CGR6889 | DPL0182 | | 2.92 | | 7.62 | | 1.88 | | 0.13 | | 0.07 | | -0.08 | 0.01 |
|  | 25 | 9 | SWU19848 | CGR6864 | | 2.82 | | 0.12 | | 5.98 | | -0.02 | | 0.09 | | 0.07 | -0.16 |
| FS | 1 | 123 | SWU11374 | NAU3384 | | 2.53 | | 1.64 | | 0.61 | | 0.14 | | -0.09 | | -0.03 | 0.12 |
|  | 5 | 114 | **SWU17715** | **Gh388** | | 3.72 | | 3.11 | | 0.01 | | 0.20 | | 0.01 | | -0.01 | -0.01 |
|  | 9 | 153 | NAU3966 | SWU15157 | | 3.23 | | 2.62 | | 0.00 | | -0.18 | | -0.01 | | 0.01 | 0.00 |
|  | 14 | 60 | SWU14224 | DPL0565 | | 4.06 | | 3.44 | | 0.58 | | -0.21 | | -0.11 | | 0.09 | 0.02 |
|  | 17 | 125 | CGR5871 | SWU12876 | | 2.53 | | 1.81 | | 0.06 | | -0.15 | | 0.04 | | -0.03 | -0.01 |
|  | 18 | 108 | NAU748 | SWU22192 | | 4.24 | | 3.66 | | 0.24 | | -0.21 | | -0.02 | | 0.07 | -0.06 |
|  | 21 | 146 | JESPR154 | SWU14431a | | 2.85 | | 2.26 | | 0.07 | | -0.17 | | 0.04 | | 0.00 | -0.04 |
|  | 21 | 172 | **BNL3171** | **CGR5808** | | 3.95 | | 3.47 | | 0.09 | | -0.21 | | -0.04 | | 0.04 | 0.00 |
|  | 22 | 2 | SWU21646 | SWU21585 | | 4.64 | | 1.38 | | 1.83 | | 0.13 | | -0.19 | | 0.18 | 0.01 |
| FE | 4 | 0 | SWU18881 | NAU2701 | | 3.14 | | 2.83 | | 1.36 | | 0.01 | | -0.01 | | 0.00 | 0.01 |
|  | 5 | 114 | **SWU17715** | **Gh388** | | 2.86 | | 2.67 | | 0.27 | | 0.01 | | 0.00 | | 0.00 | 0.00 |
|  | 9 | 151 | **NAU3966** | **SWU15157** | | 3.05 | | 3.50 | | 0.13 | | -0.01 | | 0.00 | | 0.00 | 0.00 |
|  | 11 | 7 | NAU5428 | Gh256 | | 2.82 | | 2.69 | | 0.48 | | -0.01 | | -0.01 | | 0.00 | 0.01 |
|  | 22 | 2 | SWU21646 | SWU21585 | | 2.80 | | 0.20 | | 2.73 | | 0.00 | | -0.01 | | 0.01 | 0.00 |
| FM | 5 | 14 | **SWU20913** | **Gh260** | | 2.50 | | 1.37 | | 0.30 | | -0.03 | | 0.02 | | -0.01 | -0.01 |
|  | 7 | 13 | NAU1357 | SWU10067 | | 2.80 | | 1.51 | | 0.32 | | -0.03 | | 0.02 | | -0.01 | -0.01 |
|  | 7 | 30 | SWU10064 | NAU3181 | | 2.52 | | 2.17 | | 0.18 | | -0.04 | | 0.00 | | -0.01 | 0.01 |
|  | 9 | 0 | Gh111 | Gh27 | | 3.37 | | 2.93 | | 0.16 | | 0.04 | | 0.01 | | 0.00 | -0.01 |
|  | 9 | 74 | NAU1282 | CGR6771 | | 2.64 | | 1.93 | | 0.47 | | 0.03 | | -0.01 | | 0.02 | -0.01 |
|  | 10 | 43 | Gh144 | DPL0707 | | 2.95 | | 2.54 | | 0.15 | | 0.04 | | 0.01 | | 0.00 | -0.01 |
|  | 14 | 102 | **PGML1368** | **PGML1568** | | 3.55 | | 2.82 | | 0.05 | | 0.04 | | 0.01 | | 0.00 | -0.01 |
|  | 16 | 74 | SWU10266 | DC40065 | | 4.12 | | 3.56 | | 0.39 | | 0.05 | | 0.02 | | 0.00 | -0.02 |
|  | 18 | 117 | **NAU748** | **SWU22192** | | 3.59 | | 2.74 | | 0.01 | | 0.04 | | 0.00 | | 0.00 | 0.00 |
|  | 19 | 6 | NAU5330 | Gh72 | | 3.62 | | 2.75 | | 0.30 | | 0.04 | | -0.01 | | 0.02 | -0.01 |
|  | 19 | 114 | **PGML4342** | **SWU14431b** | | 3.20 | | 0.72 | | 1.72 | | -0.02 | | 0.04 | | -0.04 | -0.01 |
|  | 22 | 4 | SWU21646 | SWU21585 | | 3.27 | | 2.11 | | 0.75 | | -0.04 | | 0.02 | | -0.03 | 0.01 |
|  | 27 | 70 | ICR11205 | DPL0847a | | 2.89 | | 2.27 | | 0.27 | | 0.04 | | 0.02 | | -0.01 | -0.01 |
