# Supplementary material for: QTLs Analysis and Validation for Fiber Quality Traits Using Maternal Backcross Population in Upland Cotton
Source: Front Plant Sci. 2017 Dec 22;8:2168. doi: 10.3389/fpls.2017.02168 (PMC5744017; doi:10.3389/fpls.2017.02168)
Supplement: Supplementary file 6 [file Table6.DOC]

**TABLE S6 | Epistatic effect QTLs and environmental interactions detected for fiber quality traits in BC population using two-locus analysis**

| **Traits** | **Ch*i*** | **Flanking markers** | | **Ch*j*** | **Flanking markers** | | **LOD** | **V(AA)%** | **V(AAE)%** | **Effect value** | | | |
| --- | --- | --- | --- | --- | --- | --- | --- | --- | --- | --- | --- | --- | --- |
|  |  |  | |  |  | |  |  |  | **AA** | **AAE1** | **AAE2** | **AAE3** |
| FL | 9 | Gh158 | DC40407 | 10 | ICR00093 | ICR07050 | 5.24 | 3.92 | 0.12 | 0.15 | -0.04 | 0.01 | 0.03 |
|  | 11 | CER0098 | CGR5421 | 11 | BNL3442b | ICR01810 | 5.32 | 3.12 | 0.58 | -0.14 | -0.08 | 0.07 | 0.01 |
|  | 2 | SWU11950 | TMB1268 | 16 | Gh56 | NAU5120 | 5.75 | 3.20 | 1.07 | 0.14 | 0.11 | -0.10 | -0.01 |
|  | 1 | CGR5663 | NAU2343 | 18 | DC40150 | ICR02849 | 6.05 | 4.21 | 0.40 | 0.17 | 0.07 | -0.07 | 0.00 |
|  | 14 | SWU14224 | DPL0565 | 19 | DC40122 | NAU833a | 5.59 | 3.59 | 0.42 | 0.16 | 0.08 | -0.04 | -0.04 |
|  | 5 | **PGML1917** | **SWU17715** | 20 | SWU20700 | CGR5548 | 5.49 | 3.83 | 0.34 | 0.16 | 0.06 | -0.02 | -0.04 |
|  | 6 | ICR00143 | CGR5108 | 20 | DPL0319 | HAU1378 | 5.06 | 3.02 | 0.61 | 0.14 | 0.05 | -0.09 | 0.04 |
|  | 19 | **SWU17782** | **DPL0056** | 21 | SWU16651 | SWU16645 | 5.54 | 3.36 | 0.67 | 0.15 | 0.07 | -0.08 | 0.02 |
|  | 1 | SWU11191 | BNL2827b | 21 | BNL3171 | CGR5808 | 5.80 | 4.04 | 0.24 | 0.16 | 0.06 | -0.02 | -0.03 |
|  | 18 | NAU748 | SWU22192 | 26 | SWU17432 | SWU17395 | 5.52 | 3.98 | 0.11 | 0.16 | 0.03 | 0.00 | -0.03 |
| FU | 3 | SWU12732 | SWU12783 | 7 | SWU10785 | CER0036 | 5.66 | 1.57 | 2.73 | 0.11 | 0.17 | -0.16 | 0.00 |
|  | 1 | SWU10986 | NAU2218 | 8 | HAU3177 | NAU4064 | 7.36 | 1.51 | 3.57 | -0.11 | -0.16 | 0.21 | -0.05 |
|  | 9 | SWU15157 | SWU14934 | 14 | SWU14224 | DPL0565 | 5.56 | 3.50 | 0.70 | 0.16 | 0.00 | -0.08 | 0.08 |
|  | 3 | SWU12783 | SWU12819 | 14 | CIR228 | BNL2485 | 5.07 | 2.07 | 1.19 | -0.12 | -0.12 | 0.12 | 0.01 |
|  | 16 | SWU10266 | DC40065 | 17 | ICR03391 | SWU12838a | 5.29 | 3.50 | 0.63 | -0.16 | -0.10 | 0.09 | 0.02 |
|  | 1 | CGR5663 | NAU2343 | 18 | DC40150 | ICR02849 | 5.05 | 2.15 | 2.45 | 0.12 | 0.16 | -0.13 | -0.03 |
|  | 15 | **DPL0182** | **SWU11691** | 18 | DC40150 | ICR02849 | 5.33 | 0.81 | 3.50 | 0.08 | 0.20 | -0.17 | -0.03 |
|  | 15 | DPL0182 | SWU11691 | 21 | Gh451 | SWU16489 | 5.97 | 1.93 | 2.52 | -0.12 | -0.18 | 0.14 | 0.04 |
|  | 6 | ICR03206 | NAU896 | 21 | SWU16138 | BNL1053 | 5.14 | 1.10 | 2.24 | 0.09 | -0.02 | -0.13 | 0.16 |
|  | 5 | CGR5025 | NBRI0694 | 21 | BNL3171 | CGR5808 | 6.29 | 2.64 | 1.89 | 0.14 | 0.10 | -0.16 | 0.06 |
|  | 11 | CGR6580 | SWU15972 | 22 | SWU21533 | DPL0562 | 5.43 | 1.98 | 1.96 | 0.12 | 0.14 | -0.16 | 0.02 |
|  | 1 | SWU0077 | HAU1417 | 24 | BNL1521 | HAU2504 | 5.21 | 1.98 | 1.61 | 0.12 | 0.15 | -0.12 | -0.03 |
|  | 19 | NAU1042 | NAU3437 | 26 | SWU17395 | DC30107 | 6.11 | 2.84 | 1.75 | 0.14 | 0.13 | -0.12 | -0.01 |
|  | 7 | NAU1357 | SWU10067 | 26 | SWU18488 | SWU18672 | 5.88 | 3.60 | 0.42 | -0.16 | -0.08 | 0.11 | -0.03 |
|  | 6 | ICR00143 | CGR5108 | 28 | NBRI0014 | SWU12107 | 5.58 | 0.27 | 3.86 | -0.03 | -0.15 | -0.09 | 0.23 |
| FS | 4 | SWU21617 | SWU11855 | 5 | SWU13378 | SWU17846 | 5.51 | 2.07 | 1.88 | -0.17 | -0.21 | 0.04 | 0.18 |
|  | 5 | CGR5025 | NBRI0694 | 14 | ICR12037 | CGR5675 | 5.19 | 3.32 | 0.45 | -0.22 | -0.09 | 0.10 | -0.01 |
|  | 3 | CER0028 | Gh663 | 15 | DC40183 | DC40175 | 5.31 | 3.64 | 0.44 | -0.22 | 0.05 | 0.06 | -0.11 |
|  | 13 | DPL0894 | SWU10800 | 17 | NAU3765 | SWU14627 | 5.20 | 2.74 | 1.02 | 0.20 | 0.12 | -0.16 | 0.04 |
|  | 3 | CER0028 | Gh663 | 18 | SWU21800 | CIR099 | 5.47 | 3.29 | 0.52 | 0.21 | -0.03 | -0.08 | 0.12 |
|  | 2 | SWU12025 | SWU11889 | 19 | DC40122 | NAU833a | 6.28 | 3.78 | 0.87 | -0.24 | -0.15 | 0.11 | 0.04 |
|  | 17 | SWU12818 | CGR5576 | 19 | SWU17897 | CGR5539 | 5.53 | 4.02 | 0.28 | -0.24 | 0.03 | 0.05 | -0.08 |
|  | 10 | HAU0635 | NAU2139 | 21 | SWU16488 | SWU16138 | 5.65 | 3.89 | 0.10 | 0.23 | 0.05 | -0.02 | -0.03 |
|  | 4 | SWU12672 | HAU1332 | 21 | SWU0830 | HAU2004 | 5.03 | 2.97 | 0.53 | 0.21 | 0.07 | -0.13 | 0.06 |
|  | 5 | **PGML1917** | **SWU17715** | 22 | SWU21586 | PGML1712 | 5.46 | 3.71 | 0.26 | 0.23 | 0.08 | -0.06 | -0.02 |
|  | 14 | CIR228 | BNL2485 | 25 | CGR6864 | SWU19815 | 5.38 | 2.84 | 0.82 | 0.20 | 0.10 | -0.14 | 0.04 |
|  | 13 | DPL0894 | SWU10800 | 25 | SWU19815 | BNL3594 | 5.57 | 3.63 | 0.61 | 0.22 | -0.03 | -0.09 | 0.12 |
|  | 17 | NAU3765 | SWU14627 | 25 | SWU19129 | PGML2858 | 6.90 | 4.03 | 0.52 | -0.24 | -0.06 | 0.12 | -0.06 |
|  | 18 | **NAU748** | **SWU22192** | 26 | SWU17432 | SWU17395 | 5.30 | 3.62 | 0.33 | 0.22 | 0.07 | 0.01 | -0.08 |
|  | 19 | NAU5330 | Gh72 | 26 | SWU0598 | SWU18698 | 5.15 | 3.71 | 0.16 | -0.23 | -0.07 | 0.05 | 0.02 |
|  | 9 | NAU5474 | Gh158 | 30 | BNL243 | CER0168 | 5.04 | 3.59 | 0.01 | -0.22 | -0.02 | 0.01 | 0.01 |
|  | 14 | ICR12037 | CGR5675 | 30 | CER0168 | SWU21718 | 5.52 | 3.89 | 0.18 | -0.23 | 0.01 | 0.05 | -0.06 |
| FE | 3 | CER0028 | Gh663 | 15 | CGR6889 | DPL0182 | 8.99 | 7.07 | 0.06 | -0.02 | 0.00 | 0.00 | 0.00 |
|  | 17 | SWU14627 | CGR5871 | 21 | CGR5808 | HAU0423 | 5.07 | 3.63 | 0.37 | -0.02 | 0.00 | 0.01 | 0.00 |
|  | 21 | CGR5806 | DPL0777 | 26 | SWU17251 | C2_0135 | 5.68 | 4.39 | 0.00 | -0.02 | 0.00 | 0.00 | 0.00 |
| FM | 1 | ICR03724 | ICR03725 | 3 | SWU12765 | NAU3839 | 5.12 | 1.56 | 2.03 | 0.03 | 0.05 | -0.02 | -0.03 |
|  | 3 | CER0028 | Gh663 | 6 | ICR00143 | CGR5108 | 6.26 | 3.92 | 0.73 | 0.05 | 0.01 | 0.02 | -0.03 |
|  | 1 | NAU6367 | MUSS422 | 8 | DC20094 | HAU1470b | 5.34 | 1.59 | 1.71 | -0.03 | -0.04 | 0.00 | 0.04 |
|  | 7 | Gh474 | SWU10785 | 9 | SWU15157 | SWU14934 | 5.41 | 3.38 | 0.76 | -0.05 | -0.03 | 0.02 | 0.02 |
|  | 5 | CGR5025 | NBRI0694 | 15 | DPL0182 | SWU11691 | 7.12 | 4.52 | 0.51 | -0.06 | -0.02 | -0.01 | 0.03 |
|  | 13 | SWU22374 | HAU2857 | 18 | SWU22281 | SWU21800 | 5.74 | 4.03 | 0.43 | -0.05 | -0.02 | 0.00 | 0.02 |
|  | 6 | ICR03206 | NAU896 | 19 | TMB0107 | NAU3217 | 5.01 | 3.22 | 0.64 | -0.05 | -0.03 | 0.02 | 0.01 |
|  | 12 | DPL0732 | Gh631 | 21 | SWU16651 | SWU16645 | 7.93 | 5.79 | 0.47 | 0.06 | -0.01 | 0.03 | -0.02 |
|  | 16 | SWU10038 | ICR00016 | 21 | CGR5808 | HAU0423 | 5.32 | 3.67 | 0.40 | -0.05 | -0.02 | 0.00 | 0.02 |
|  | 16 | PGML1709 | SWU10627 | 23 | PGML4186 | NAU3100 | 5.10 | 2.18 | 2.18 | 0.04 | 0.05 | -0.02 | -0.03 |
|  | 21 | CGR5217 | BNL3442a | 23 | PGML4186 | NAU3100 | 5.56 | 3.63 | 0.67 | -0.05 | -0.01 | -0.02 | 0.03 |
|  | 3 | SWU12732 | SWU12783 | 24 | HAU2504 | SWU13736 | 5.07 | 2.63 | 1.20 | -0.04 | -0.04 | 0.01 | 0.03 |
|  | 1 | SWU0077 | HAU1417 | 25 | HAU1382 | SWU19848 | 6.16 | 3.47 | 1.46 | 0.05 | -0.01 | 0.04 | -0.03 |
|  | 23 | SWU14807 | PGML4185 | 25 | HAU1382 | SWU19848 | 5.79 | 4.13 | 0.59 | 0.05 | 0.00 | 0.03 | -0.02 |
|  | 20 | CGR6154 | SWU20246 | 25 | BNL3594 | DPL0282 | 5.20 | 2.14 | 1.81 | -0.04 | -0.05 | 0.02 | 0.02 |
|  | 26 | SWU17395 | DC30107 | 26 | BNL2495 | DPL0491 | 5.26 | 3.34 | 0.00 | -0.08 | 0.00 | -0.04 | 0.04 |
|  | 24 | SWU13758 | CGR5423 | 26 | SWU18488 | SWU18672 | 5.14 | 2.17 | 0.97 | -0.04 | -0.01 | -0.02 | 0.04 |
|  | 2 | SWU11889 | SWU11887 | 26 | SWU18672 | SWU18681 | 5.33 | 2.97 | 1.10 | 0.04 | 0.04 | 0.00 | -0.03 |
|  | 7 | SWU10205 | HAU1483a | 27 | SWU10994 | HAU1001 | 5.09 | 3.76 | 0.08 | 0.05 | 0.01 | 0.00 | -0.01 |
|  | 21 | HAU0423 | CGR5806 | 27 | **CGR6857** | **ICR11205** | 5.30 | 3.40 | 0.52 | 0.05 | 0.03 | 0.00 | -0.02 |
|  | 20 | CER0167 | SWU20064 | 29 | BNL3261 | CGR5111 | 5.81 | 4.14 | 0.34 | 0.05 | -0.01 | 0.02 | -0.01 |
|  | 19 | **NAU5330** | **Gh72** | 31 | CGR6254 | SWU16676 | 5.76 | 2.99 | 1.29 | 0.05 | 0.01 | 0.03 | -0.04 |
