# Supplementary material for: QTLs Analysis and Validation for Fiber Quality Traits Using Maternal Backcross Population in Upland Cotton
Source: Front Plant Sci. 2017 Dec 22;8:2168. doi: 10.3389/fpls.2017.02168 (PMC5744017; doi:10.3389/fpls.2017.02168)
Supplement: Supplementary file 7 [file Table7.DOC]

**TABLE S7 | Summary statistics on fiber quality trait data of RIL and BC populations in six environments**

| **Trait** | **Env.^1^** | **BC^2^** |  |  |  | **RIL** |  |  |  | **Parents** |  |  |  |  |
| --- | --- | --- | --- | --- | --- | --- | --- | --- | --- | --- | --- | --- | --- | --- |
|  |  | **Mean** | **Range** | **CV%^3^** |  | **Mean** | **Range** | **CV%** |  | **♀** | **♂** | **Xinza 1** | **MPH%^4^** | **CK^5^** |
| Fiber length (mm) | 2012E1 | 28.24 | 26.56-29.87 | 2.24 |  | 28.61 | 26.24-31.23 | 3.35 |  | 27.91 | 27.73 | 29.16 | 4.82 | 29.68 |
|  | 2012E2 | 26.96 | 24.56-29.46 | 3.40 |  | 26.53 | 23.83-29.17 | 4.44 |  | 26.16 | 25.74 | 26.41 | 1.77 | 29.67 |
|  | 2012E4 | 30.35 | 29.09-32.13 | 1.94 |  | 30.30 | 27.82-32.62 | 2.93 |  | 29.75 | 30.45 | 30.68 | 1.93 | 30.10 |
|  | 2015E1 | 29.51 | 27.20-31.80 | 2.88 |  | 29.42 | 27.10-32.70 | 3.52 |  | 28.38 | 28.27 | 27.93 | -1.39 | 29.40 |
|  | 2015E2 | 31.01 | 28.85-33.20 | 2.37 |  | 30.85 | 28.05-33.45 | 3.53 |  | 30.63 | 30.00 | 30.88 | 1.86 | 31.18 |
|  | 2015E3 | 30.91 | 29.10-33.20 | 2.51 |  | 30.77 | 27.85-34.05 | 3.53 |  | 30.30 | 29.85 | 29.73 | -1.15 | 30.53 |
| Fiber uniformity | 2012E1 | 84.36 | 81.95-86.85 | 1.11 |  | 84.10 | 81.60-86.30 | 0.98 |  | 83.95 | 84.98 | 83.35 | -1.32 | 85.40 |
|  | 2012E2 | 83.64 | 79.75-86.25 | 1.25 |  | 83.31 | 79.60-85.75 | 1.30 |  | 83.08 | 82.40 | 84.15 | 1.70 | 85.68 |
|  | 2012E4 | 86.63 | 84.95-88.15 | 0.72 |  | 86.49 | 84.25-88.30 | 0.97 |  | 85.30 | 86.65 | 86.65 | 0.79 | 86.70 |
|  | 2015E1 | 84.19 | 81.45-86.35 | 1.19 |  | 84.02 | 80.60-86.55 | 1.19 |  | 83.58 | 85.30 | 84.43 | -0.01 | 84.93 |
|  | 2015E2 | 85.99 | 83.45-87.85 | 0.78 |  | 85.81 | 83.55-87.40 | 0.89 |  | 86.30 | 85.73 | 86.25 | 0.27 | 86.08 |
|  | 2015E3 | 85.09 | 82.30-87.05 | 0.94 |  | 84.95 | 82.15-87.00 | 1.08 |  | 86.50 | 85.48 | 86.00 | 0.01 | 85.70 |
| Fiber strength (cN/tex) | 2012E1 | 29.75 | 27.30-32.50 | 3.24 |  | 30.51 | 25.75-34.25 | 4.66 |  | 30.20 | 29.53 | 30.23 | 1.22 | 31.98 |
|  | 2012E2 | 28.62 | 26.30-31.70 | 3.34 |  | 27.56 | 24.10-30.70 | 4.35 |  | 27.88 | 27.25 | 28.23 | 2.41 | 29.60 |
|  | 2012E4 | 29.51 | 27.80-31.15 | 2.32 |  | 29.29 | 26.80-32.20 | 3.42 |  | 29.48 | 29.10 | 29.23 | -0.20 | 27.98 |
|  | 2015E1 | 30.04 | 26.80-34.30 | 4.28 |  | 30.12 | 26.15-33.85 | 5.60 |  | 29.68 | 30.47 | 28.25 | -6.07 | 30.67 |
|  | 2015E2 | 30.43 | 27.80-34.20 | 3.32 |  | 30.49 | 27.05-36.20 | 4.74 |  | 30.45 | 29.57 | 30.05 | 0.13 | 31.93 |
|  | 2015E3 | 31.23 | 28.15-33.80 | 3.84 |  | 31.18 | 27.10-35.60 | 5.24 |  | 30.20 | 30.18 | 29.45 | -2.45 | 29.70 |
| Fiber elongation | 2012E1 | 6.68 | 6.40-6.95 | 1.74 |  | 6.92 | 6.35-7.35 | 2.55 |  | 6.78 | 6.83 | 6.88 | 1.10 | 7.03 |
|  | 2012E2 | – | – | – |  | – | – | – |  | – | – | – | – | – |
|  | 2012E4 | 6.86 | 6.60-7.05 | 1.25 |  | 6.90 | 6.50-7.20 | 1.97 |  | 6.95 | 6.93 | 7.00 | 0.86 | 6.80 |
|  | 2015E1 | 6.96 | 6.70-7.20 | 1.18 |  | 6.94 | 6.65-7.25 | 1.63 |  | 6.80 | 6.93 | 6.88 | 0.22 | 6.87 |
|  | 2015E2 | 6.98 | 6.80-7.20 | 0.94 |  | 6.96 | 6.70-7.15 | 1.21 |  | 6.98 | 6.93 | 7.00 | 0.65 | 6.98 |
|  | 2015E3 | 7.02 | 6.75-7.20 | 1.21 |  | 7.00 | 6.70-7.3 | 1.56 |  | 6.98 | 6.95 | 6.88 | -1.22 | 6.90 |
| Micronaire | 2012E1 | 4.34 | 3.83-4.98 | 5.23 |  | 4.37 | 3.21-5.26 | 8.35 |  | 4.57 | 4.61 | 4.49 | -2.18 | 5.07 |
|  | 2012E2 | 4.52 | 3.89-5.19 | 5.32 |  | 4.49 | 3.69-5.27 | 6.06 |  | 4.56 | 4.57 | 4.52 | -0.99 | 5.05 |
|  | 2012E4 | 5.18 | 4.53-5.57 | 3.73 |  | 4.97 | 3.88-5.74 | 7.04 |  | 5.38 | 4.81 | 5.21 | 2.26 | 5.05 |
|  | 2015E1 | 4.79 | 3.80-5.50 | 6.29 |  | 4.62 | 3.40-5.40 | 8.35 |  | 4.90 | 4.53 | 4.98 | 5.62 | 5.07 |
|  | 2015E2 | 4.91 | 4.10-5.70 | 5.69 |  | 4.85 | 3.95-5.80 | 7.31 |  | 5.15 | 4.87 | 4.78 | -4.59 | 5.28 |
|  | 2015E3 | 5.47 | 4.90-5.90 | 3.23 |  | 5.39 | 4.50-5.95 | 5.33 |  | 5.58 | 5.53 | 5.48 | -1.35 | 5.40 |

*^1^ Environment in 2012 and 2015, E1, Handan, E2, Cangzhou, E3, Wuhan, E4, Xiangyang. ^2^ RIL: the recombinant inbred line population; BC: the maternal backcross population. ^3^ Coefficient of variation. ^4^ Mid-parent heterosis (%). ^5^ Competition control of ‘Ruiza 816’ in E1 and E2 in* [*Yangtze valley*](http://www.baidu.com/link?url=cRov71-Rm01mLuHSk-6mju6bjFsgxFg39zlzKY6dSM6Tc86WoHPXcwHIf4p10NlAD-VF_UJOm5Prs5eqC98HDX8MLzVE1c6zcj2F7lXi_22HMyKuMd07JKtSP9b6lSlN)*, and ‘Ezamian 10’ in E3 and E4 in Yellow River valley.*
