# Supplementary material for: QTLs Analysis and Validation for Fiber Quality Traits Using Maternal Backcross Population in Upland Cotton
Source: Front Plant Sci. 2017 Dec 22;8:2168. doi: 10.3389/fpls.2017.02168 (PMC5744017; doi:10.3389/fpls.2017.02168)
Supplement: Supplementary file 8 [file Table8.DOC]

**TABLE S8 | Summary on QTL mapping results and stable QTLs for fiber quality traits in intraspecific segregation populations**

| **Fiber quality traits^a^** | **Cross** | **Population (size)** | **Linkage group** | **No. of QTL** | **No. of stable QTL** | **Reference** |
| --- | --- | --- | --- | --- | --- | --- |
| FL, FS, FE, FU, FM, M, SFI, R, Y | ZMS12×8891 | RIL (180) | 17 | 48 | 11 | Wang et al., 2006 |
| FU, FS, SL2.5 | Yumian-1×T586 | RIL (270) | 1 | 8 | 0 | Wan et al., 2007 |
| FL, FS, FE, FM | 7235×TM-1 | RIL (258) | 6 | 11 | 11 | Shen et al., 2006 |
| FE, FS, FM, SL2.5, SL50, M, P, WF, WT | HS-46×MARCABUCAG8US-1-88 | RIL (188) | 13 | 33 | 0 | Wu et al., 2009 |
| FL, FS, FE, FU, FM | (Yumian-1×CRI35)×(Yumian-1×7235) | CP (172) | 69 | 63 | 27 | Zhang et al., 2012 |
| FL, FS, FE, FU, FM | sGK9708×0-153 | F_2_(250) F_2:3_ (196), RIL (196) | 9, 14 | 20, 40 | 50 | Sun et al., 2012 |
| FL, FS, FE, FU, FM | GX1135×GX100-2 | F_2_(256) F_2:3_ (173), F_2:4_ (173) |  | 39 | 16 | Liang et al,, 2013 |
| FL, FS,FE,FM, SFI | MD90ne × MD52ne | F_2_ (378) | 3 | 4 | 3 | Islam et al., 2014 |
| FL, FS, FM, FE, FU, SFI | Acala Prema × 86-1 | RIL (180) | 6 | 53 | 10 | Ning et al., 2013 |
| FL, FS, FE, FU, FM | CCRI 35×Yumian 1 | RIL (180) | 23 | 59 | 11 | Tan et al., 2014 |
| FL, FS, FE, FU, FM | Yumian 1×7235 | RIL (180) | 23 | 62 | 17 | Tang et al., 2015 |
| FL, FS, FE, FM, FU | Yumian 1×AcalaMaxxa, CA3084, TAM94L-25 | F_2_, F_2:3_, F_2:4_ (180) | 24 | 77 | 13 | Shao et al., 2014 |
| FL, FS, FE, FU, FM | 0-153×sGK9708 | RIL(196) | 1 | 37 | 17 | Zhang et al., 2015b |
| FL, FS, FE, FU, FM | GX1135×GX100-2, VGX100-2 | RIL (177), BC (177) | 19, 12 | 111 | 62 | Shang et al., 2016a |
| FL, FS, FE, FU, FM | 0–153×sGK9708 | RIL (196) | 24 | 165 | 37 | Jamshed et al., 2016 |
| FL, FS, FE, FU, FM | HS-46×MARCABUCAG8US-1-88 | RIL (188) | 21 | 71 | 9 | Li et al., 2016 |
| FL, FS, FE, FU, FM | DH962×Jimian5 | BC (178, 178) | 22 | 76 | 8 | Wang et al., 2016 |

^a^2.5% span length (SL2.5), 50% span length (SL50), fiber maturity (M), perimeter (P), weight fitness (WF), and wall thickness (WT), short fiber index (SFI), reflectance(R), yellowness(Y). The stable QTL refers to QTL detected in more than one environment or population. CP, composite cross population.
